# Hydrogen peroxide attenuates rhinovirus-induced anti-viral interferon secretion in sinonasal epithelial cells

**DOI:** 10.3389/fimmu.2023.1086381

**Published:** 2023-02-13

**Authors:** Sang Hag Lee, Mun Soo Han, Tae Hoon Lee, Da Bin Lee, Jae Hyung Park, Seung Hyeok Lee, Tae Hoon Kim

**Affiliations:** Department of Otorhinolaryngology-Head & Neck Surgery, College of Medicine, Korea University, Seoul, Republic of Korea

**Keywords:** rhinovirus, interferon, chronic rhinosinusitis with nasal polyps, sinonasal epithelial cells, viperin, Mx, OAS, Nrf2

## Abstract

**Background:**

Altered innate defense mechanisms, including an imbalance between oxidants and antioxidants release, have been implicated in the pathogenesis of chronic rhinosinusitis (CRS). The aim of this study is to investigate whether oxidative stress may attenuate the secretion of anti-viral interferons in human sinonasal mucosa.

**Methods:**

The levels of H_2_O_2_ in nasal secretion were increased in patients with CRS with nasal polyps, compared with that of CRS patients without nasal polyps and control subjects. Normal sinonasal epithelial cells derived from healthy subjects were cultured under an air-liquid interface. The cultured cells were infected with rhinovirus 16 (RV 16) or treated with poly (I: C), TLR3 agonist, after being pretreated with an oxidative stressor, H_2_O_2_ or antioxidant, N-acetylcysteine (NAC). Thereafter, the expression levels of type I (IFN-β) and type III (IFN-λ1 and λ2) interferons and interferon-stimulated genes (ISGs) were evaluated with RT-qPCR, ELISA, and western blot.

**Results:**

The data showed that the production of type I (IFN-β) and type III (IFN-λ1 and λ2) interferons and ISGs was upregulated in cells infected with RV 16 or treated with poly (I: C). However, their up-regulated expression was attenuated in cells pretreated with H_2_O_2,_ but not inhibited in cells pretreated with NAC. In line with these data, the up-regulated expression of TLR3, RIG-1, MDA5, and IRF3 was reduced in cells pretreated with H_2_O_2,_ but not attenuated in cells treated with NAC. Furthermore, cells transfected with Nrf2 siRNA showed decreased secretion of anti-viral interferons whereas sulforaphane treatment enhanced the secretory capacity of antiviral interferons.

**Conclusions:**

These results suggest that the production of RV16-induced antiviral interferons may be attenuated by oxidative stress.

## Introduction

Chronic rhinosinusitis (CRS) refers to persistent and heterogeneous chronic inflammation in the sinonasal mucosa lining the nasal and paranasal sinus cavities. Multiple etiological factors, including bacterial biofilms and staphylococcal superantigens, have been implicated in the development of CRS (1). Clinically, rhinosinusitis symptoms in patients with CRS are frequently exacerbated after viral upper respiratory infections. Respiratory viral infections caused by rhinovirus (RV) are common in CRS patients (2, 3). RV infection induces excessive mucus secretion and bacterial adhesion, and disrupts tight junction components in epithelial cells, suggesting that RV infections are a potential causative factor in the initial development, perpetuation, and acute exacerbations of CRS (4–7).

A double-stranded (ds)-RNA produced during RV infection is initially recognized through three pattern recognition receptors (PRRs) distributed in epithelial cells: toll-like receptor (TLR)-3 and the RNA helicases including melanoma differentiation-associated protein (MDA)-5, and retinoic acid-inducible gene (RIG)-1 (8–10). Stimulation of PRRs by ds-RNA results in the secretion of anti-viral interferons (IFNs) to eradicate virus spread, initiating a complex signaling pathway through protein kinase complexes (11–13).

Respiratory viral infections in the lower respiratory tract have been extensively investigated, in contrast to the sinonasal epithelial cells of the upper respiratory tract. These studies have shown that defective anti-viral interferon production in patients with chronic inflammatory disorders of lower respiratory tract enhances susceptibility to viral infections (14–17). An *in-vitro* study using bronchial epithelial cells revealed that RV-16-induced interferon production attenuated by Th2 cytokines resulted in increased rhinovirus replication, suggesting that Th2 inflammation can increase susceptibility to viral infection (18). In a previous study, the secretion of anti-viral interferons was decreased in patients with CRS, suggesting the possibility that decreased secretion may lead to deficient anti-viral innate responses in CRS (19). Stimulation with Th2 cytokines in sinonasal epithelial cells resulted in the reduction of anti-viral interferon release (19). Nevertheless, the detailed regulatory mechanisms that govern deficient anti-viral interferon release in CRS are poorly understood. Therefore, mechanisms or molecules modulating deficient anti-viral interferon release in CRS should be investigated as potential targets to attenuate the disease process.

Oxidative stress has been linked to disease severity in chronic inflammatory disorders of the lower respiratory tract including COPD (20, 21). Hydrogen peroxide (H_2_O_2_) acts as a mediator of oxidative stress and contributes to the pathogenesis of asthma and COPD. The production of H_2_O_2_ is increased in patients with COPD and asthma, suggesting that H_2_O_2_ might be a biomarker for assessing disease severity (22–24). Nuclear factor erythroid 2-related factor 2 (Nrf2) is an endogenous enhancer of cellular antioxidants and Nrf2 activation leads to the transcription of cellular antioxidants (25, 26). Therefore, Nrf2 deficiency aggravates oxidative stress and is associated with increased symptom severity in asthma and COPD (27, 28). Furthermore, it has been reported that the antioxidant, N-acetyl-L-cysteine (NAC), inhibits the progression of airway diseases associated with oxidative stress and also attenuates COPD exacerbations (29–31). Although the role of oxidants and antioxidants in sinonasal mucosal inflammation in CRS is unclear, accumulating evidence shows that the expression of cytoprotective enzymes, including heme oxygenase-1 and dual oxidases, and H_2_O_2_-producing isoforms of nicotinamide adenine dinucleotide phosphate, is increased in CRS, suggesting that oxidative stress participates in the pathophysiology of CRS (1, 32, 33). Furthermore, a recent study revealed an increased concentration of H_2_O_2_ in the nasal lavage fluid of patients with CRS (32). Nevertheless, whether oxidative stress in the sinonasal mucosa impairs the anti-viral innate immune response in CRS has not been determined yet.

Therefore, this study was to elucidate 1) whether the production of H_2_O_2_ increases in the nasal secretion of patients with CRS; 2) whether oxidative stressor, H_2_O_2,_ and antioxidant, NAC, modulate anti-viral interferon (IFN) release such as type I and type III IFNs, and IFN stimulated genes (ISGs) in sinonasal mucosa and the expression of PRRs; and 3) whether Nrf2 knockdown and activator modulate the release of anti-viral interferons in the sinonasal mucosa.

## Materials and methods

### Subjects

Patients with CRS, according to EPOS 2020 guidelines (1), were divided as follows: group I, CRS without nasal polyps (CRSsNP, n=23), and group II, CRS with nasal polyps (CRSwNP, n=26). Patients with blowout fractures (n=15) and patients with a deviated septum and hypertrophic rhinitis (n=50) were used as healthy subjects or controls (**Supplementary Table 1)**. Normal sinonasal mucosa was obtained from the uncinate processes during septoplasty in patients with a deviated septum and hypertrophic rhinitis for epithelial cell culture.

Written informed consent was obtained from all the participants. The protocol of this study was approved by the ethics committee of the University Hospital. Disease severity was assessed by computed tomography of the paranasal sinus and endoscopic findings, and severity of the symptoms was analyzed using the visual analog system, as previously described (34–36). The subjects with previous sinus surgery, asthma, allergic rhinitis, or aspirin sensitivity were excluded. None of the patients received any medication, including antibiotics, antihistamines, or steroids during the past three months.

### H_2_O_2_ measurement in nasal lavage fluid

Nasal secretions were collected by nasal lavage from control subjects with blowout fractures and patients with CRS. Briefly, 5 mL of phosphate-buffered saline (PBS) was flushed through a catheter into each nasal cavity. Thereafter, lavage fluid was collected in a sterile tube. The supernatant obtained after centrifuging the pooled lavage fluid was stored in a deep freezer until H_2_O_2_ analysis.

The concentration of H_2_O_2_ in nasal secretions was evaluated using the Amplex Red hydrogen peroxide assay kit (Invitrogen, CA, USA). In brief, a buffer solution in 100 μM Amplex Red reagent was added to nasal secretions collected by nasal lavage and then mixed with 0.2 U/mL horseradish peroxidase (HRP), adjusted to a final volume of 100 μL. The reaction of Amplex Red reagent with H_2_O_2_ resulted in the production of red fluorescent resorufin, which was evaluated using a fluorescence plate reader. BCA protein assay kit (Thermo Fisher Scientific, Mass, USA) was employed to measure the concentration of total protein contained in nasal lavage fluid.

### Culture of isolated sinonasal epithelial cells

Epithelial cells were detached from the sinonasal mucosa of control subjects by enzymatic degradation and grown in culture plates (SPL, Pocheon City, Korea) filled with bronchial epithelial cell media (BEpiCM, ScienCell Lab, CA, USA). Cells obtained at passage 2 were placed at a density of 1 × 10^6^ cells/cm^2^ on a Transwell insert (SPL) and cultivated under an air-liquid interface (ALI) in a mixture of BEpiCM and DMEM/F12. After epithelial cells reached full confluence, they were used for the experiments.

### Experimental design

To evaluate whether oxidative stress and Nrf2 signaling may modulate the secretion of anti-viral interferons in epithelial cells, experimental groups were allocated as follows: 1) normal groups (untreated and uninfected with RV-16); 2) RV-16 infection control (epithelial cells with RV-16 without pretreatment); 3) H_2_O_2_ or NAC pretreatment followed by RV-16 infection; 4) H_2_O_2_ or NAC pretreatment followed by poly (I: C); and 5) RV-16 inoculation or poly (I: C) stimulation in Nrf2-silenced cells or Nrf2-activating cells.

Pretreatment of cultured epithelial cells with H_2_O_2_ (Sigma-Aldrich, Seoul, Korea) was conducted at concentrations of 50, 100, and 150 μM, for 1 h, and pretreatment with NAC was carried out at a concentration of 5 mM for 1 h. Thereafter, the epithelial cells were inoculated with 0.5 multiplicity of infection (MOI) of RV-16 for 48 h and stimulated with poly (I: C) at a concentration of 10 μM for 24 h.

To analyze whether Nrf2 signaling may modulate the secretion of anti-viral interferons, Nrf2 siRNA was transfected into one group of epithelial cells or the pretreatment with Nrf2 activator, sulforaphane (5 μM, Sigma Aldrich, St. Louis, USA) was performed in other groups of epithelial cells for 48 h and thereafter, these cells were infected with 0.5 MOI of RV-16 for 48 h or stimulated with poly (I: C) at a concentration of 10 μM for 24 h.

Thereafter, the secretion of anti-viral interferons in harvested cells and basal media was measured using RT-qPCR, ELISA, and Western blot. Furthermore, the expression of SOD1, SOD2, RIG1, MDA5, and TLR3 mRNA transcripts and proteins was evaluated using RT-qPCR and western blot. A lactate dehydrogenase assay kit (Abcam, Cambridge, UK) was used to test the cell viability.

### RV-16 propagation and infection

Human rhinovirus 16 (ATCC VR-283PQ) was proliferated in H1HeLa cells (ATCC, Manassas, VA, USA), which were cultured in Eagle’s minimum essential medium (EMEM, Thermo Fisher Scientific) at 33°C. A cytopathic effect was evaluated by measuring the 50% tissue culture infective doses (TCID_50_).

The ALI cultures were washed with Dulbecco’s phosphate-buffered saline (DPBS) to remove mucus secreted from the cultured epithelial cells. Thereafter, cultured cells were inoculated with RV-16 at an MOI of 0.5 and incubated for 5 h in Minimum Essential Medium (MEM) at 33°C with 5% CO_2_ in the air. Thereafter, the cultured cells were rinsed thrice with DPBS to remove the non-attached virus, and then, apical and basal spaces of Transwell inserts (SPL) were supplemented with ALI medium, incubating for 48 h at 33°C.

To analyze the replication rate of RV 16 during the experimental period, basal media and cells harvested at 48h after inoculation were kept in a deep freezer at -75°C. Viral RNA was extracted from cultured cells using a QIAamp Viral RNA Mini Kit (Qiagen, Ontario, Canada). HRV 16 Genesig standard kit^®^ (Primerdesign™ LTD., Chandler’s Ford, UK) was used for the analysis of RV 16 RNA levels by RT-qPCR. Standard curves for the quantification of RV 16 were made by RV16 RNA standards included in the kit.

The data for the antiviral mediators were evaluated in each group of cultured cells after RV 16 replication is detected and the replication rates were presented in **Supplementary Figures 1**-**5**.

### Transfection of Nrf2 small interfering RNA into sinonasal epithelial cells

We transfected cultured epithelial cells with small interfering RNA (siRNA) directed towards Nrf2 with Lipofectamine 2000 (Invitrogen, Carlsbad, CA, USA) for 48 h. Human Nrf2 siRNA sequences were constructed as follows: GAGACUACCAUGGUUCCA and UUGGAACCAUGGUAGUCUC, and the blocking effect of Nrf2 siRNA was tested by RT-qPCR and western blotting.

### RT-qPCR

Total RNA was extracted from the cultured cells using a Qiazol lysis reagent (QIAZEN Inc, CA, USA). The quantification of total RNA was carried out with a NanoDrop 2000c Spectrophotometer (Thermo Fisher Scientific). Extracted RNA (2 μg) was used for cDNA synthesis with a reaction fluid containing Oligo dT primers (GenDEPOT). RT-qPCR was performed using specific primers of each gene (**Supplementary Table 2**) and SYBR-green (Takara Bio, Shiga, Japan).

### Western blot analysis

Cultured epithelial cells after stimulation were rinsed with DPBS and proteins were extracted using radio-immunoprecipitation assay (RIPA) buffer (GenDEPOT). Denatured proteins (25 μg) were fractionated on SDS-PAGE and the gels were transferred onto a polyvinylidene difluoride membrane (Bio-Rad, MA, USA). Thereafter the membranes were rinsed and probed with the primary antibodies in the refrigerator overnight at 4 °C; the primary antibodies employed for Western blots were anti-β actin (1:5000) which was obtained from Santa Cruz, USA, anti-viperin, anti-OAS, anti-Mx, anti-TLR3, anti-RIG1, anti-MDA5, anti-SOD1, anti-SOD2, anti-IRF3, and anti-phospho-IRF3 which were obtained from Cell Signaling Technology and used at a dilution of 1:1000.

### Determination of anti-viral interferons levels by ELISA

Basal media was used to analyze the release of interferon (IFN)-β, -λ1, and -λ2 with ELISA (R&D Systems).

### Statistical analysis

Statistical analyses were carried out using the SPSS package (ver 16.0.0 for Windows; SPSS Inc., Chicago, IL, USA). One-way analysis of variance was used for the comparison of the endoscopic scores, Sino-Nasal Outcome Test-20, and the age differences among the four groups, and the statistical difference of the data obtained by RT-qPCR, ELISA, and Western blot. Multiple comparisons were conducted by the Kruskal-Wallis test with Bonferroni posthoc test. Data are expressed as the mean ± standard errors (SEM). Significant differences were set at P < 0.05.

## Results

### The concentration of H_2_O_2_ in nasal secretions

**Figure 1:**
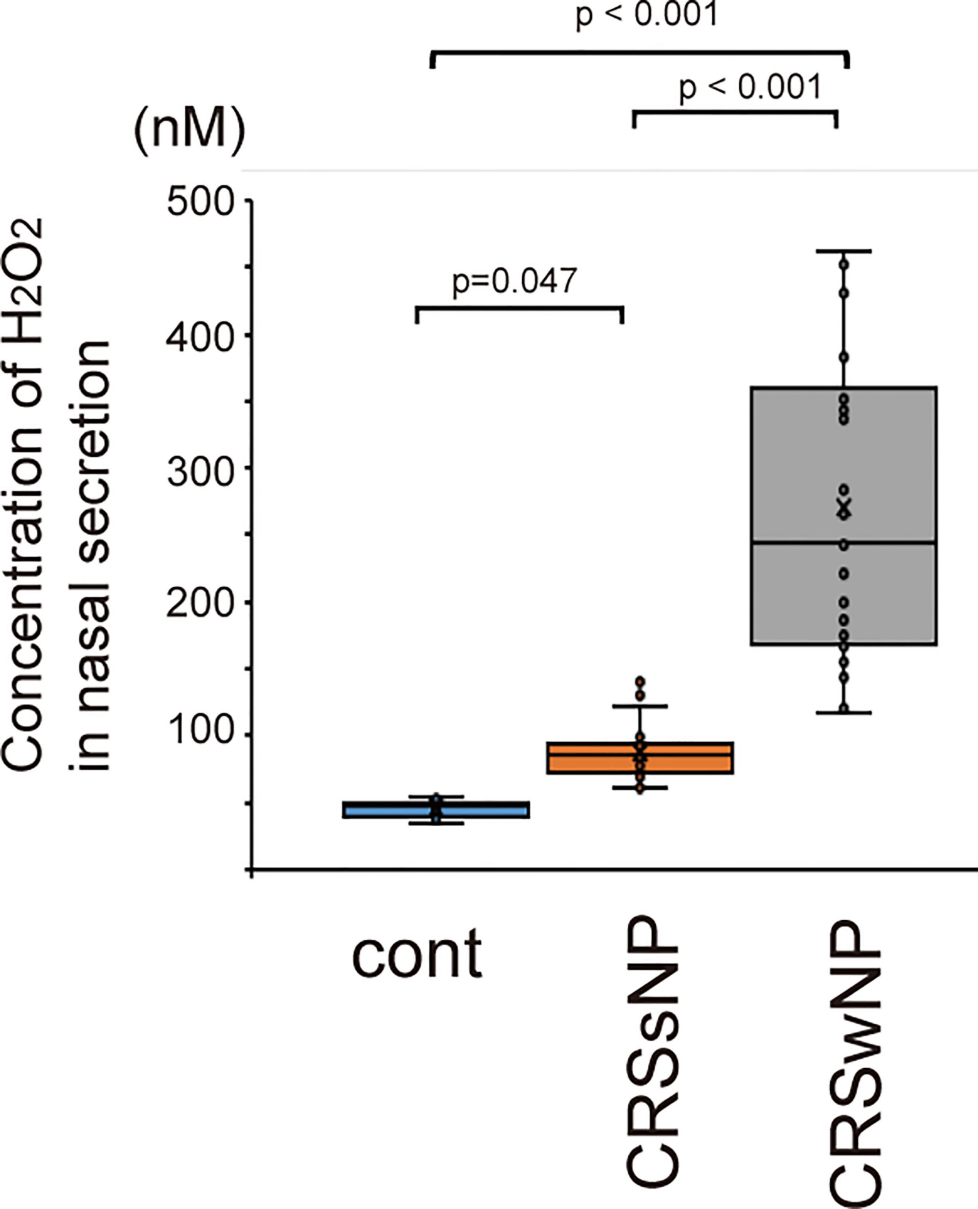
The concentration of H_2_O_2_ in nasal secretion of normal control (n=15) and patients with CRSsNP (n=23) and CRSwNP (n=26). CRSsNP indicates chronic rhinosinusitis without nasal polyps. CRswNP means chronic rhinosinusitis with nasal polyps. Data are mean ± SEM.

**Figure 1** shows the H_2_O_2_ concentrations in the nasal secretion of healthy controls, patients with CRSsNP and patients with CRSwNP. The concentration of H_2_O_2_ was 35.15 ± 1.45 nM in nasal secretions from normal healthy subjects, 95.71 ± 3.18 nM in patients with CRSsNP, and 270.73 ± 113.62 nM in patients with CRSwNP. The concentration of H_2_O_2_ in the nasal lavage fluid of patients with CRS was higher than that in normal healthy subjects (**Figure 1**). Furthermore, the H_2_O_2_ concentration in CRSwNP was higher than that in CRSsNP (**Figure 1**).

### Expression of superoxide dismutase is attenuated by H_2_O_2_ pretreatment in sinonasal epithelial cells

**Figure 2:**
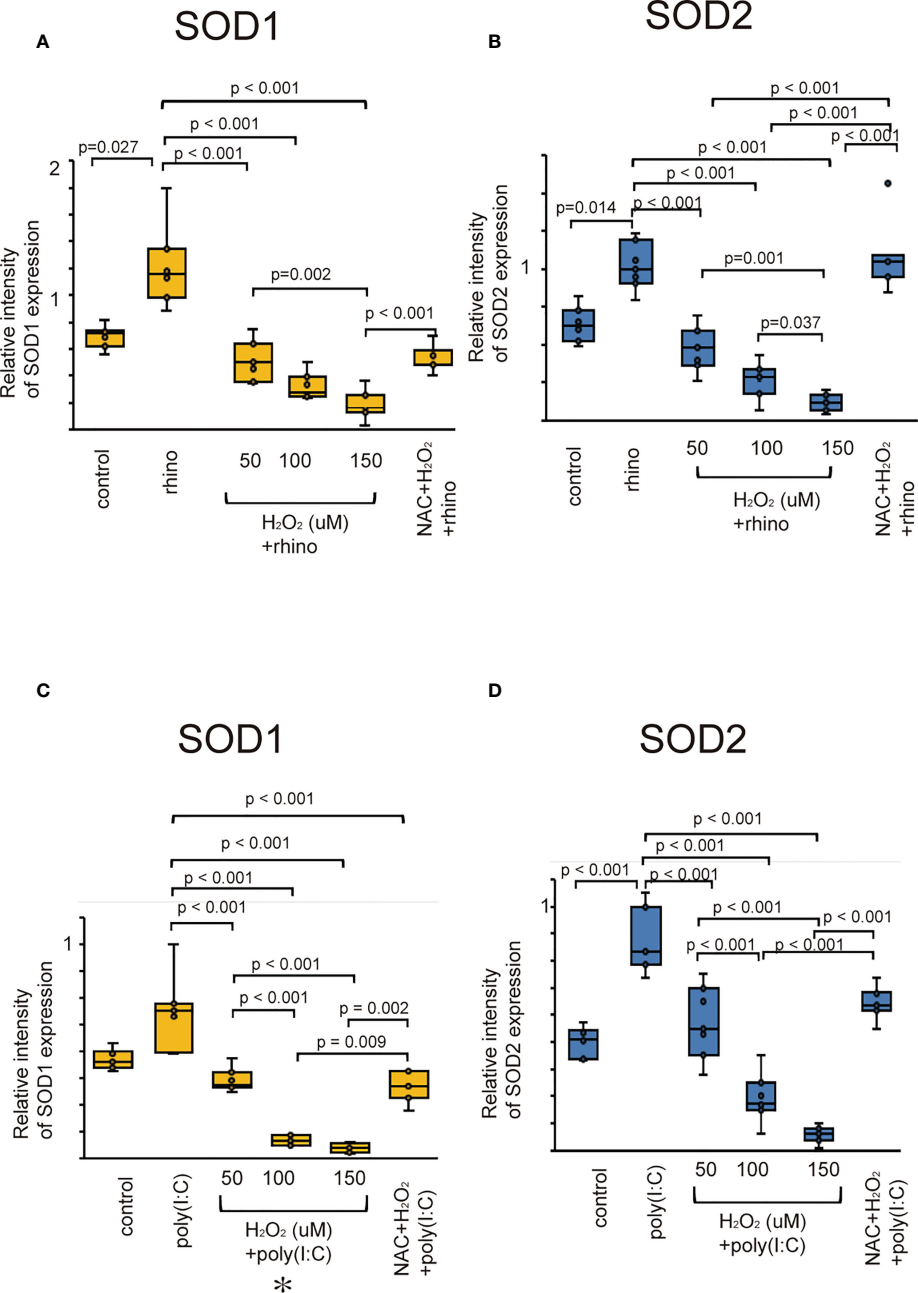
The expression levels of SOD1 **(A, C)** and SOD2 (**B, D)** in cultured sinonasal epithelial cells pretreated with H_2_O_2_ at 50, 100, and 150 uM and then followed by RV 16 infection **(A, B)** and poly (I:C) treatment **(C, D)**, which were evaluated by western blot. Data are mean ± SEM from 7 different epithelial donors. Control indicates non-treated normal epithelial cells. Rhino indicates epithelial cells infected with RV 16. H_2_O_2_ +rhino indicates the epithelial cells pretreated with H_2_O_2_ at 50, 100, and 150 uM followed by RV 16 infection. NAC+ H_2_O_2_ +rhino indicates the cells which were pretreated with NAC at 5 mM for 1 h and then followed by H_2_O_2_ treatment at 100 uM and subsequently infected with RV 16. Poly (I:C) indicates epithelial cells treated with poly (I:C). H_2_O_2_ +poly (I:C) indicates the epithelial cells pretreated with H_2_O_2_ at 50, 100, and 150 uM followed by poly (I:C) treatment. NAC+ H_2_O_2_ +poly (I:C) indicates the cells which were pretreated with NAC at 5 mM for 1 h and then followed by H_2_O_2_ treatment at 100 uM and subsequently treated with poly (I:C). Rhino indicates RV 16. NAC indicates N-acetyl-L-cysteine.

Superoxide dismutase (SOD) is an essential antioxidant enzyme that modulates oxidative homeostasis (37). We evaluated whether rhinovirus infection induces the expression of SOD1 and SOD2 in sinonasal epithelial cells. Cells infected with RV-16 showed increased protein levels of SOD1 and SOD2 compared to control cells (**Figures 2A, B**). However, their upregulation was attenuated in cells pretreated with H_2_O_2_ at 50, 100, and 150 uM (**Figures 2A, B**). Furthermore, down-regulation of SOD1 and SOD2 protein levels by H_2_O_2_ pretreatment was inhibited in cells treated with NAC (**Figures 2A, B**). In line with data from RV-16-infected cells, the expression levels of SOD1 and SOD2 protein were increased when treated with poly (I: C) and their up-regulation was inhibited by H_2_O_2_ pretreatment (**Figures 2C, D**). Furthermore, the down-regulation of SOD1 and SOD2 levels by H_2_O_2_ pretreatment was reversed by NAC pretreatment (**Figures 2C, D**). These findings were confirmed by the results of RT-qPCR (**Supplementary Figure 6**).

### Effect of pretreatment with H_2_O_2_ and NAC on the production of anti-viral interferons

**Figure 3:**
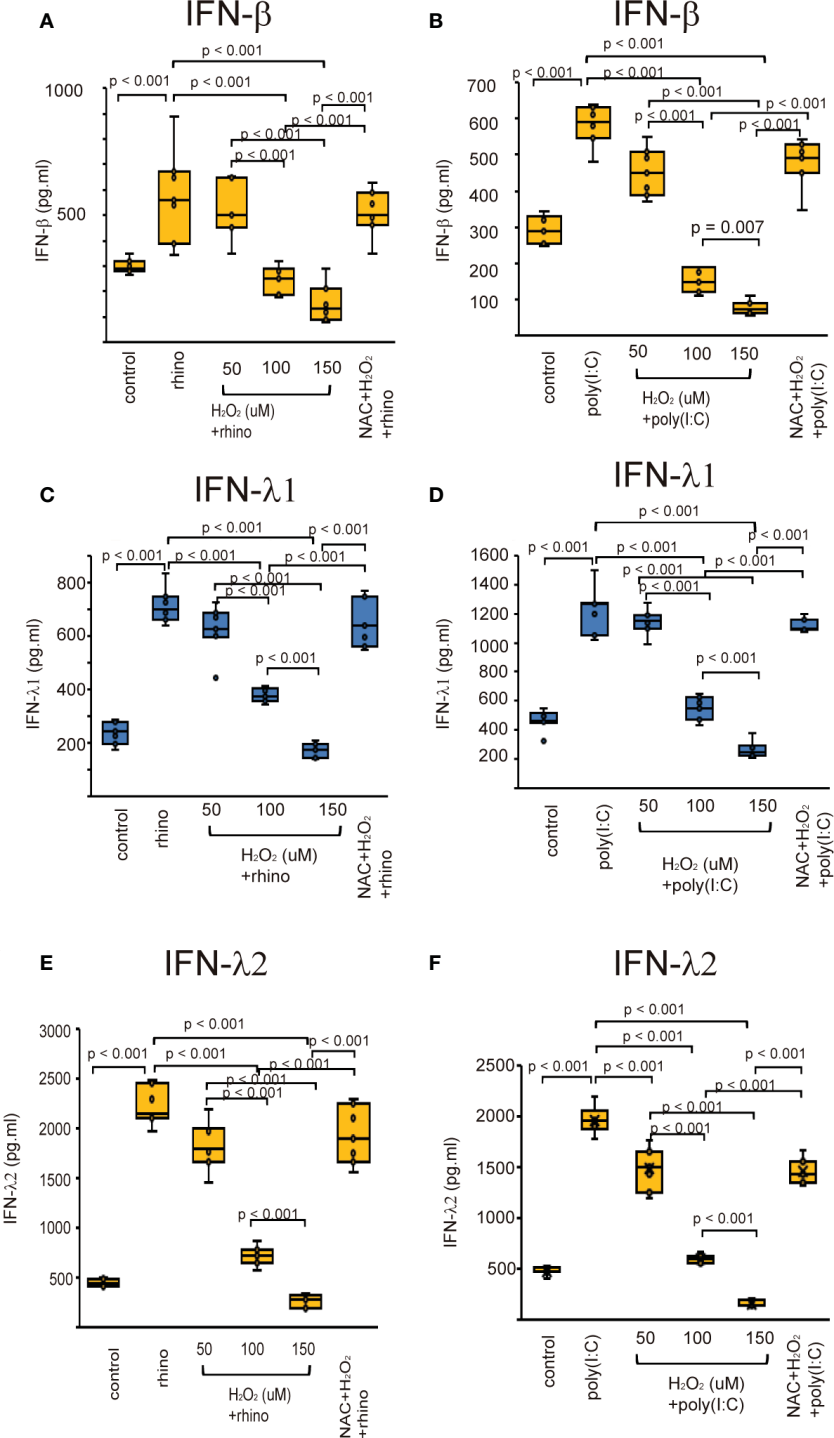
The concentration of IFN-β **(A, B)**, IFN-λ1 **(C, D)**, and IFN-λ2 **(E, F)** in basal media of cultured cells pretreated with H_2_O_2_ at 50, 100, and 150 uM and then, followed by RV 16 infection **(A, C, E)** or poly (I: C) treatment **(B, D, F)** which were evaluated with ELISA. Data are mean ± SEM from 7 different epithelial donors. Control indicates non-treated normal epithelial cells. Rhino indicates epithelial cells infected with RV 16. H_2_O_2_ + rhino indicates the epithelial cells pretreated with H_2_O_2_ at 50, 100, and 150 uM followed by RV 16 infection. NAC+ H_2_O_2_ +rhino indicates the cells which were pretreated with NAC at 5 mM for 1 h and then followed by H_2_O_2_ treatment at 100 uM and subsequently infected with RV 16. Poly (I: C) indicates epithelial cells treated with poly (I: C). H_2_O_2_ + poly (I: C) indicates the epithelial cells pretreated with H_2_O_2_ at 50, 100, and 150 uM followed by poly (I: C) treatment. NAC+ H_2_O_2_ + poly (I: C) indicates the cells which was pretreated with NAC at 5 mM for 1 h and then followed by H_2_O_2_ treatment at 100 uM and subsequently treated with poly (I: C).

The effect of H_2_O_2_ on the production of type I and III IFNs, and ISGs was evaluated in H_2_O_2-_pretreated cells after infection with RV-16 and treatment with poly (I: C). The production of type I and III IFNs were up-regulated when being exposed to RV-16 or poly (I: C) compared to the control cells (**Figure 3**, **Supplementary Figure 2**). However, their up-regulation was inhibited by pretreatment with H_2_O_2_ in a dose-dependent manner (**Figures 3**, **Supplementary Figure 2**). To clarify the involvement of oxidative stress in the secretion of anti-viral interferons, we investigated the effect of the antioxidant, NAC on the down-regulation of anti-viral interferons. Pretreatment with NAC inhibited the H_2_O_2_-induced suppression of anti-viral interferon secretion (**Figures 3**, **Supplementary Figure 2**). The data of RT-qPCR also showed the secretion of type I and III IFNs increased by RV-16 infection and poly (I: C) treatment, which was attenuated by the pretreatment with H_2_O_2_ (**Supplementary Figure 2**). However, H_2_O_2_-induced down-regulation was significantly attenuated by NAC pretreatment (**Supplementary Figure 7**).

**Figure 4:**
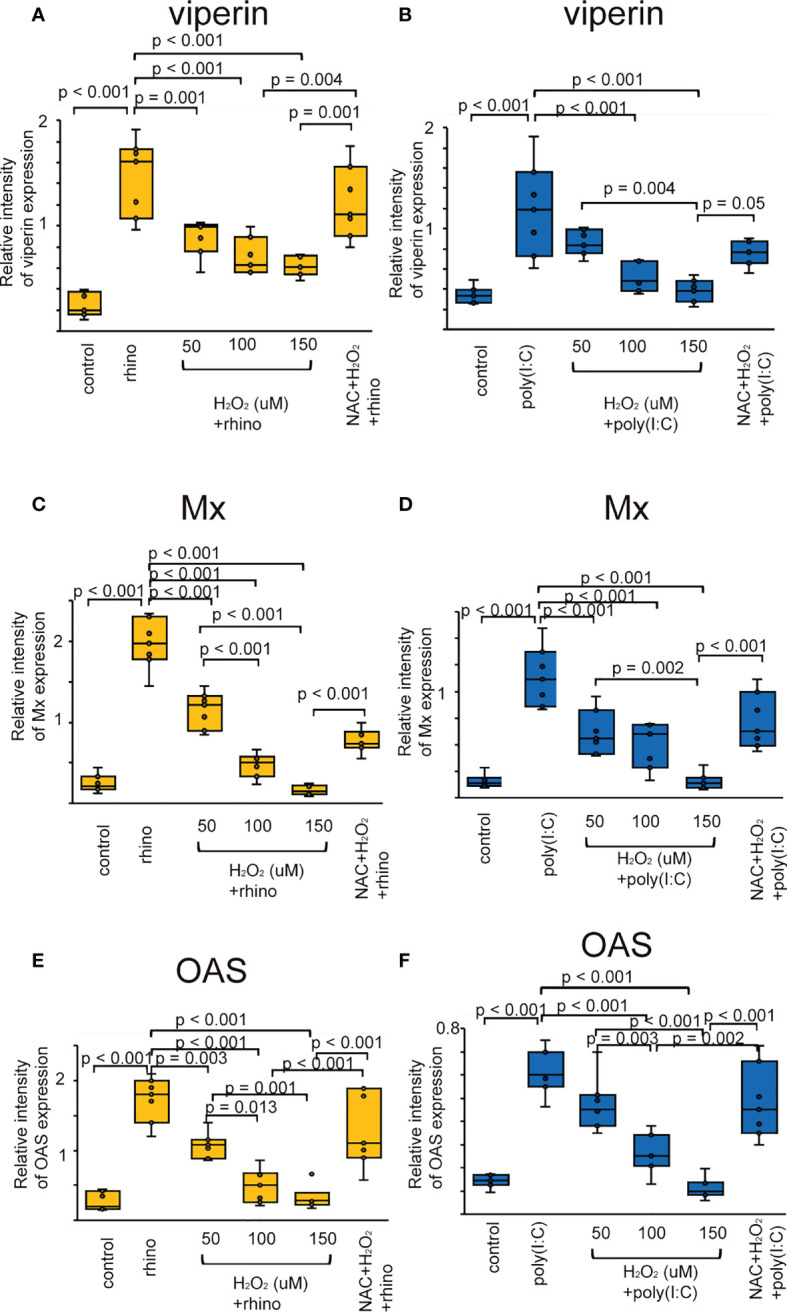
The expression level of viperin **(A, B)**, Mx **(C, D)**, and OAS **(E, F)** proteins in sinonasal epithelial cells pretreated with H_2_O_2_ at 50, 100, and 150 uM and then, followed by RV 16 infection (**A, C, E)** or poly (I: C) treatment **(B, D, F)** which were evaluated with western blot. Data are mean ± SEM from 7 different epithelial donors. Control indicates non-treated normal epithelial cells. Rhino indicates epithelial cells infected with RV 16. H_2_O_2_ + rhino indicates the epithelial cells pretreated with H_2_O_2_ at 50, 100, and 150 uM followed by RV 16 infection. NAC+ H_2_O_2_ +rhino indicates the cells which were pretreated with NAC at 5 mM for 1 h and then followed by H_2_O_2_ treatment at 100 uM and subsequently infected with RV 16. Poly (I: C) indicates epithelial cells treated with poly (I: C). H_2_O_2_ + poly (I: C) indicates the epithelial cells pretreated with H_2_O_2_ at 50, 100, and 150 uM followed by poly (I: C) treatment. NAC+ H_2_O_2_ + poly (I: C) indicates the cells which was pretreated with NAC at 5 mM for 1 h and then followed by H_2_O_2_ treatment at 100 uM and subsequently treated with poly (I: C).

The augmented expression of ISG proteins observed in RV-16 infected and poly (I: C) treated cells was also inhibited by pretreatment with H_2_O_2_ (**Figure 4**). This attenuation was significantly subsided in the cells pretreated with NAC (**Figure 4**). These findings were verified by the data of RT-qPCR (**Supplementary Figure 8**).

### Effect of pretreatment with H_2_O_2_ and NAC on expression of TLR3, RIG-1, and MDA5

**Figure 5:**
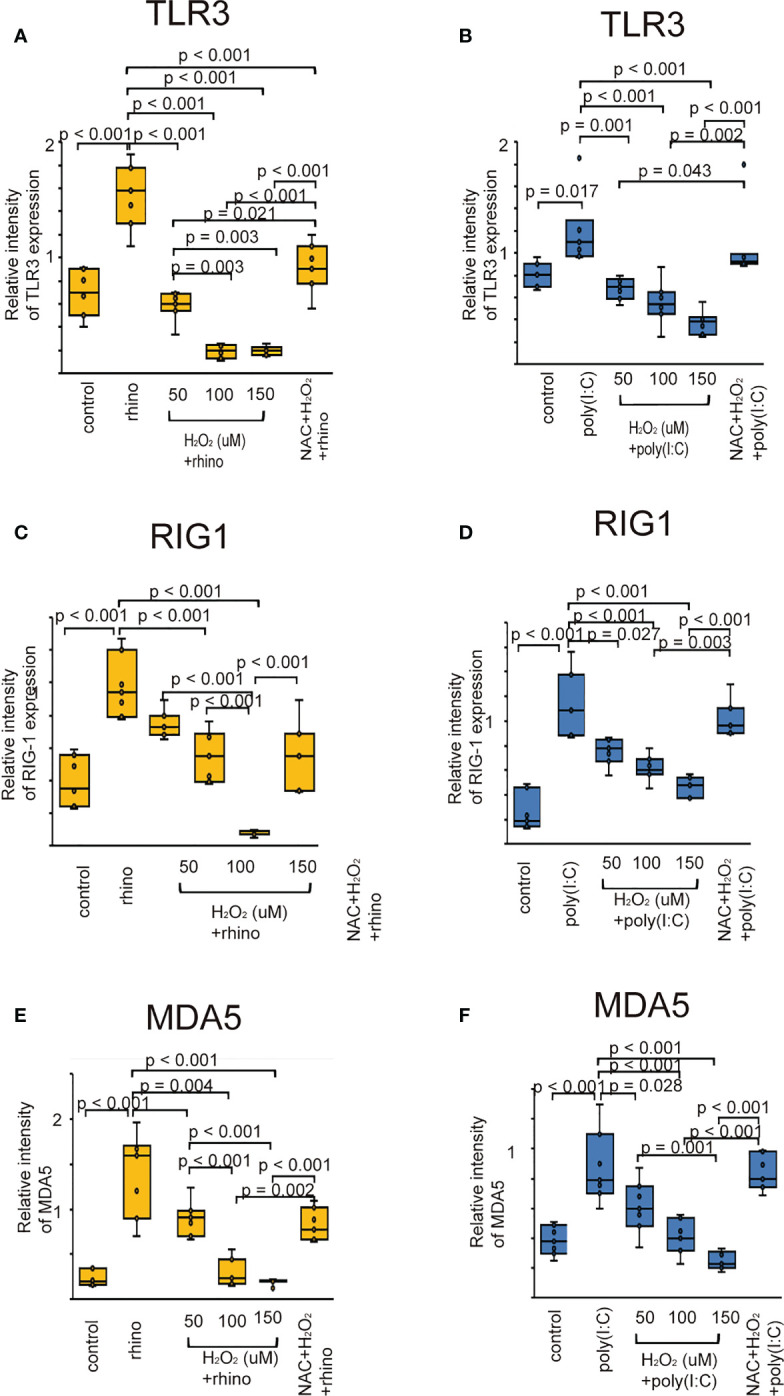
The expression level of TLR3 **(A, B)**, RIG-I **(C, D)**, and MDA5 proteins **(E, F)** in sinonasal epithelial cells pretreated with H_2_O_2_ at 50, 100, and 150 uM and then, followed by RV 16 infection **(A, C, E)** or poly (I: C) treatment **(B, D, F)** which were evaluated with western blot. Data are mean ± SEM from 7 different epithelial donors. Control indicates non-treated normal epithelial cells. Rhino indicates epithelial cells infected with RV 16. H_2_O_2_ + rhino indicates the epithelial cells pretreated with H_2_O_2_ at 50, 100, and 150 uM followed by RV 16 infection. NAC+ H_2_O_2_ +rhino indicates the cells which were pretreated with NAC at 5 mM for 1 h and then followed by H_2_O_2_ treatment at 100 uM and subsequently infected with RV 16. Poly (I: C) indicates epithelial cells treated with poly (I: C). H_2_O_2_ + poly (I: C) indicates the epithelial cells pretreated with H_2_O_2_ at 50, 100, and 150 uM followed by poly (I: C) treatment. NAC+ H_2_O_2_ + poly (I: C) indicates the cells which was pretreated with NAC at 5 mM for 1 h and then followed by H_2_O_2_ treatment at 100 uM and subsequently treated with poly (I: C).

Considering the evidence that RV infection enhances the innate immune response through PRRs (8–10), we explored whether oxidative stress attenuates the expression of TLR3, RIG-1, and MDA5. Their protein levels were elevated in epithelial cells infected with RV 16 or stimulated with poly (I: C) (**Figure 5**). However, their upregulation was attenuated by pretreatment with H_2_O_2_ in a dose-dependent manner (**Figure 5**). Their downregulatory effect induced by H_2_O_2_ pretreatment was inhibited in cells pretreated with NAC (**Figure 5**). These findings were verified by the data of RT-qPCR (**Supplementary Figure 9**).

### Effect of pretreatment with H_2_O_2_ and NAC on the expression of phospho-IRF3

**Figure 6:**
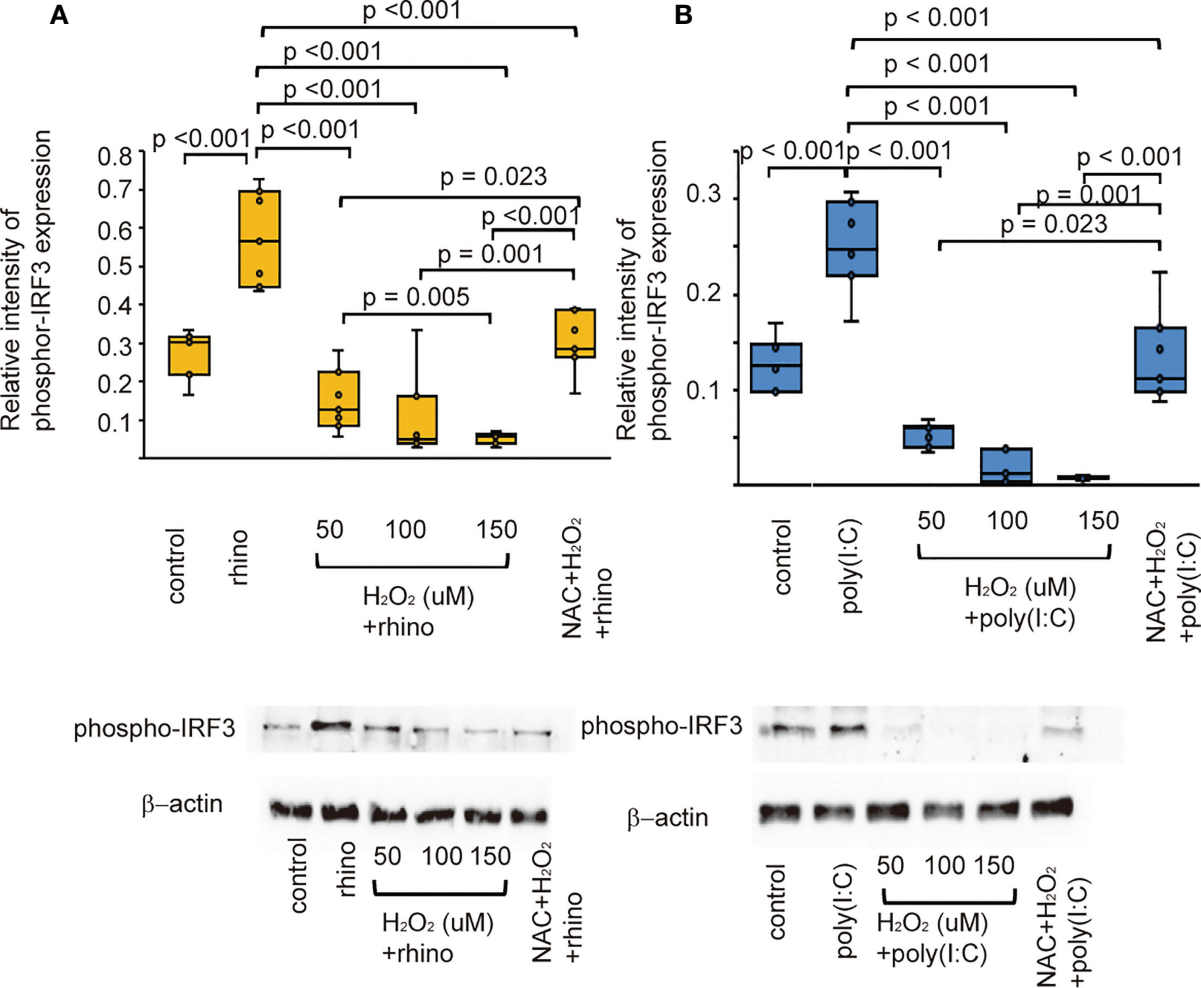
The expression level of phosphor-IRF3 proteins in sinonasal epithelial cells pretreated with H_2_O_2_ at 50, 100, and 150 uM and then, followed by RV 16 infection **(A)** and poly (I: C) treatment **(B)** which were evaluated with western blot. Data are mean ± SEM from 7 different epithelial donors. Control indicates non-treated normal epithelial cells. Rhino indicates epithelial cells infected with RV 16. H_2_O_2_ + rhino indicates the epithelial cells pretreated with H_2_O_2_ at 50, 100, and 150 uM followed by RV 16 infection. NAC+ H_2_O_2_ +rhino indicates the cells which were pretreated with NAC at 5 mM for 1 h and then followed by H_2_O_2_ treatment at 100 uM and subsequently infected with RV 16. Poly (I: C) indicates epithelial cells treated with poly (I: C). H_2_O_2_ + poly (I: C) indicates the epithelial cells pretreated with H_2_O_2_ at 50, 100, and 150 uM followed by poly (I: C) treatment. NAC+ H_2_O_2_ + poly (I: C) indicates the cells which was pretreated with NAC at 5 mM for 1 h and then followed by H_2_O_2_ treatment at 100 uM and subsequently treated with poly (I: C). Lower panels show representative protein bands evaluated with western blot.

Once TLR3 is activated, its downstream signaling pathway activates interferon regulatory factor-3 (IRF3) (38). Therefore, the effects of H_2_O_2_ and NAC on the expression of IRF3 were evaluated to verify the inhibitory effect of oxidative stress on the secretion of anti-viral interferon. RV-16 infected cells showed an increased expression of phospho-IRF3 compared with that of control cells (**Figure 6A**). Poly (I: C)-stimulated cells also showed similar findings (**Figure 6B**). Furthermore, its levels decreased after pretreatment with H_2_O_2_. Phospho-IRF3 levels suppressed by pretreatment with H_2_O_2_ were inhibited in cells pretreated with NAC (**Figure 6**).

### Effect of Nrf2 on the production of anti-viral interferons

**Figure 7:**
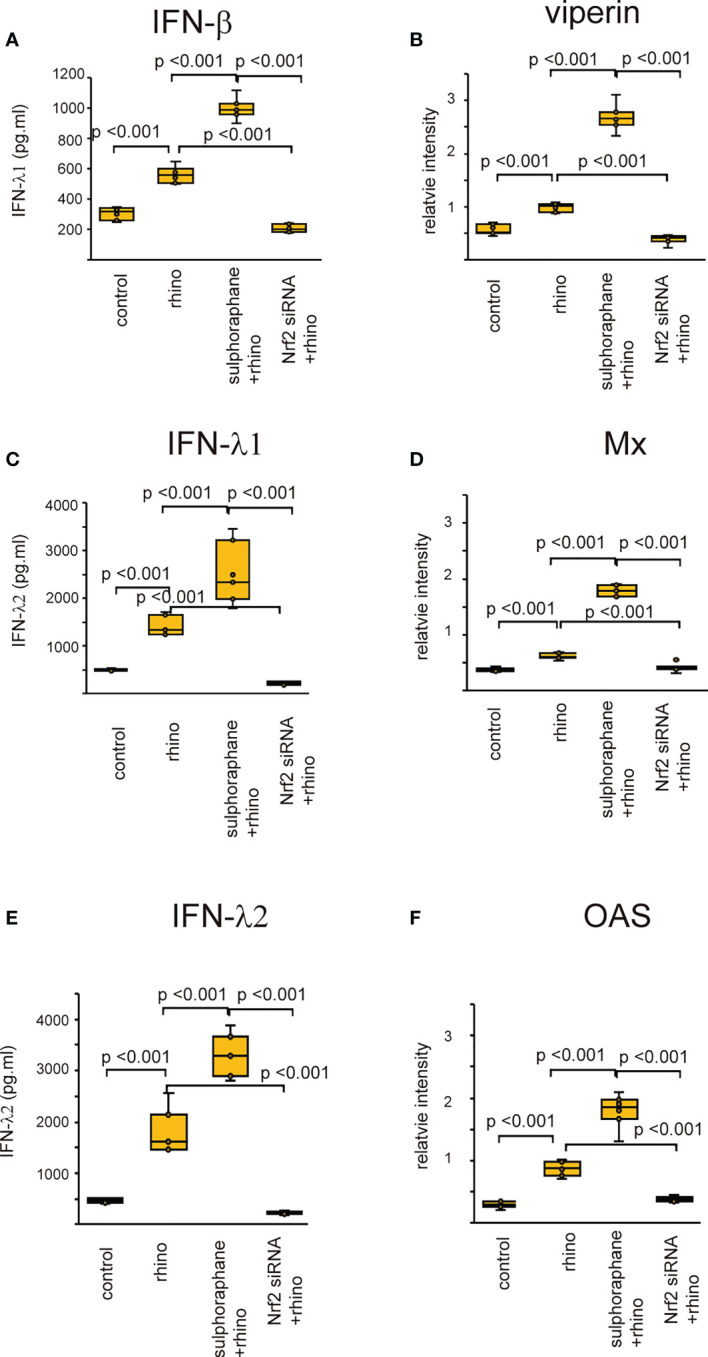
The concentration of IFN-β **(A)**, IFN-λ1 **(C)**, and IFN-λ2 **(E)** in basal media of cultured cells and the expression levels of viperin **(B)** Mx **(D)** and OAS **(F)** in cultured cells transfected with Nrf2 siRNA and pretreated with sulforaphane and then, followed by RV 16 infection which were evaluated with ELISA **(A, C, E)** and western blot **(B, D, F)**. Data are mean ± SEM from 7 different epithelial donors. Control indicates non-treated normal epithelial cells. Rhino indicates epithelial cells infected with RV 16. Sulforaphane + rhino indicates the epithelial cells pretreated with sulforaphane at 5 uM followed by RV 16 infection. Nrf2siRNA + rhino indicates the cells transfected with Nrf2 siRNA were infected with RV 16.

**Figure 8:**
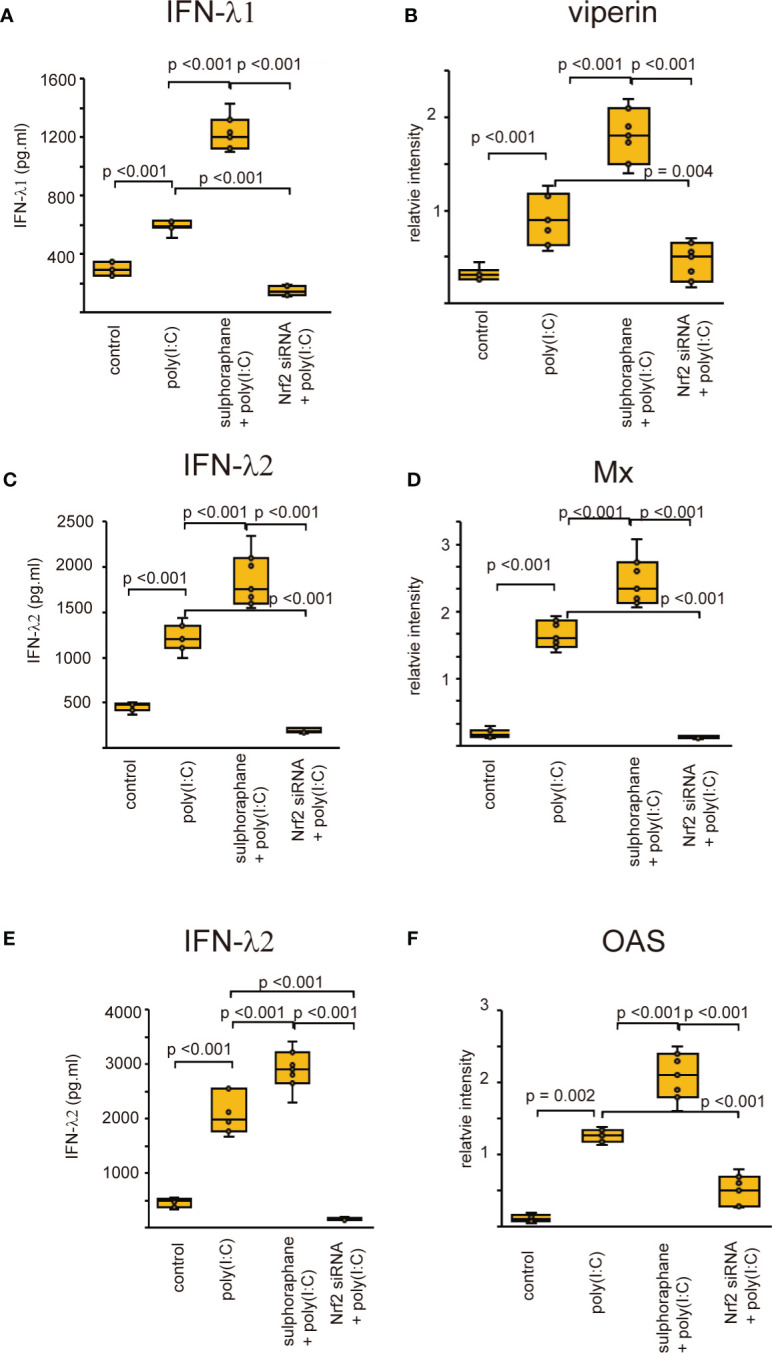
The concentration of IFN-β **(A)**, IFN-λ1 **(C)**, and IFN-λ2 **(E)** in basal media of cultured cells and the expression levels of viperin **(B)** Mx **(D)** and OAS **(F)** in cultured cells transfected with Nrf2 siRNA and pretreated with sulforaphane and then, followed by poly (I:C) treatment which were evaluated with ELISA **(A, C, E)** and western blot **(B, D, F)**. Data are mean ± SEM from 7 different epithelial donors. Control indicates non-treated normal epithelial cells. Poly (I: C) indicates epithelial cells treated with Poly (I: C). Sulforaphane + Poly (I: C) indicates the epithelial cells pretreated with sulforaphane at 5 uM followed by Poly (I: C) treatment. Nrf2siRNA + Poly (I:C) indicates the cells transfected with Nrf2 siRNA treated with Poly (I: C).

Recent studies revealed that Nrf2 is an endogenous inducer of cellular antioxidant molecules (25, 26). To explore the involvement of Nrf2 in the secretion of anti-viral interferons, we evaluated the expression of anti-viral interferons in cells transfected with Nrf2 siRNA or pretreated with Nrf2 activator, sulforaphane. The levels of antiviral interferons including IFNs and ISGs were increased in cells infected with RV-16 or stimulated with poly (I: C) (**Figures 7**, **8**, **Supplementary Figures 10**, **11**). Pretreatment with the Nrf2 activator, sulforaphane, potentiated their up-regulated expression enhanced by RV-16 infection or poly (I: C) stimulation (**Figures 7**, **8**). However, the augmentation was significantly inhibited in groups transfected with Nrf2 siRNA (**Figures 7**, **8**). Furthermore, RT-qPCR showed similar results. (**Supplementary Figures 10**, **11**).

## Discussion

Oxidative stress is implicated in the severity of symptoms in patients with chronic inflammatory disorders of the lower airway, such as asthma and COPD (20, 21). In the present study, the concentration of H_2_O_2_ increased in the nasal lavage fluid of patients with CRS. Based on these data, we used H_2_O_2_ as an oxidative stressor to elucidate the effects of oxidative stress on RV-induced anti-viral interferon secretion. Sinonasal epithelial cells showed up-regulated expression of SOD1 and SOD2 after inoculation with RV-16 or stimulation with poly (I: C). Furthermore, the expression levels of anti-viral interferons, ISGs, and PRRs, including TLR3, RIG-1, and MDA5, were also increased in cells infected with RV-16 or treated with poly (I: C). However, H_2_O_2_ pretreatment suppressed the up-regulation of anti-viral interferons and PRRs in a dose-dependent manner. These suppressive effects were inhibited in cells pretreated with the anti-oxidant, NAC. Furthermore, the secretion of anti-viral interferons was up-regulated in sulforaphane-treated cells whereas their secretion was attenuated in Nrf2-silenced cells. These results suggest that oxidative stress attenuates the secretion of anti-viral interferons in the sinonasal mucosa.

Oxidative stress is enhanced when reactive oxygen species (ROS) are excessively produced in the body and is closely associated with inflammatory processes. ROS, responsible for oxidative stress in the respiratory system, consist of hydroxyl radicals, superoxide anions, and H_2_O_2_ (39). Elevated H_2_O_2_ levels are a dominant feature of oxidative stress and hallmarks of inflammation (40).. H_2_O_2_ is known to be the most stable ROS *in vivo* and acts as a key mediator of inflammation (41). Therefore, exposure to H_2_O_2_ is widely used to induce oxidative damage in cellular models. Several studies have shown that ROS contribute to the inflammatory process in CRSwNP, demonstrating that oxidative stress participates in the pathogenesis of CRSwNP (42, 43). The levels of free radicals and malondialdehyde-thiobarbituric acid, a product of free radical-induced lipid peroxidation, are higher in nasal polyps than in normal mucosal tissues (44, 45). However, the role of H_2_O_2_ as oxidative stress in ROS production by human sinonasal epithelial cells has not been well explored. A previous study showed a higher concentration of H_2_O_2_ in the nasal lavage fluids of patients with CRSwNP than in those of patients with CRSsNP and healthy control (32). These data were confirmed by the present study, suggesting a relationship between H_2_O_2_ concentration and CRS disease severity.

SOD is an important antioxidant enzyme that converts superoxide into less reactive peroxide, and is essential for the regulation of oxidative homeostasis (37). RV infection has a definite effect on oxidative stress and enhances antioxidant expression (46, 47). In line with these results, our data demonstrated that SOD1 and SOD2 expression increases in sinonasal epithelial cells infected with RV-16 or stimulated with poly (I: C), suggesting that RV-16 infection or the stimulation of TLR3 can induce antioxidant expression in sinonasal epithelial cells. Furthermore, pretreatment with H_2_O_2_ attenuated the expression of SOD1 and SOD2 in sinonasal epithelial cells. In contrast to cells pretreated with H_2_O_2_, the decreased levels of SOD1 and SOD2 were reversed by pretreatment with the antioxidant NAC. Therefore, considering the evidence that oxidative stress contributes to the pathogenesis of CRSwNP (42, 43), these data support the clinical finding of antioxidant deficiency in CRSwNP. This suggestion is supported by results showing that H_2_O_2_-pretreated bronchial epithelial cells reduced the expression levels of SOD (48). However, the effect of viral infection on SOD expression in host cells appears to differ depending on the type of viral pathogen. Infection of cells with the influenza virus results in the induction of SOD2 without a change in SOD1 (49). In A549 cells infected with RSV, SOD1 expression was significantly decreased whereas SOD2 was increased (50).

Deficient expression of anti-viral interferons and ISGs has been observed in patients with CRS (19). However, the mechanisms enhancing the deficient release of anti-viral interferons and ISGs are largely unknown. Previous studies have shown that oxidative stress enhanced by H_2_O_2_ pretreatment attenuates antiviral-interferon secretion in respiratory epithelial cells derived from patients with asthma and COPD (48). Pretreatment of A549 cells with H_2_O_2_ prior to RSV infection downregulated the mRNA expression of IRF3 (50). Cigarette smoke extract, a known source of oxidants, inhibited the activation of IRF-3, which was enhanced by poly (I: C) in normal bronchial epithelial cell lines (BEAS-2B) (51). Influenza virus-induced secretion of anti-viral cytokines was completely attenuated in both Calu-3 cells and human lung tissues after smoke exposure, a known source of oxidants (52). Furthermore, treatment with cigarette smoke extract inhibited influenza-induced IFN-β mRNA expression (53). RIG-1 induction by the virus was inhibited by cigarette smoke extract and prevented by the antioxidant NAC (53). These data are in line with the present results demonstrating reduced secretion of type I and III IFNs and ISGs in human sinonasal epithelial cells pretreated with H_2_O_2_. Conversely, other studies have provided additional evidence that oxidative stress may contribute to reduced anti-viral interferon secretion. FOXO3a participates in the attenuation of oxidative stress by enhancing the production of antioxidant enzymes, including SOD (54–56). Similarly, pretreatment with MitoTEMPO, a mitochondrial-specific antioxidant, restored anti-viral IFN secretion following RV infection in FOXO3a knockout airway epithelial cells (57). Exposure to air pollutants resulted in impaired IFN-β expression in the airway epithelium (58).

Activation of TLR3, RIG-1, MDA5, and IRF3 results in the release of anti-viral interferon in cells infected with RNA virus (8–10, 12,). Similarly, we previously confirmed the up-regulated levels of TLR3, RIG-1, MDA5, and phospho-IRF3 in sinonasal epithelial cells when being infected with RV-16 or treated with poly (I: C) (59). However, few studies have evaluated the relationship between RV infection with TLR3, RIG-1, MDA5, phospho-IRF3, and oxidative stress. Therefore, we carried out to determine whether the expression of PRRs, including TLR3 and the activation of IRF3 are deficient in cells pretreated with H_2_O_2_. The data showed that the expression of PRRs could be modulated by oxidative stress when being infected with RV-16 or stimulated with a TLR3 agonist. Similar to our data, Menzel et al. reported that IFN-β gene expression decreased in H_2_O_2_-pretreated cells, accompanied by reduced expression of pattern recognition receptors (48). Akbarshahi et al. showed that human bronchial epithelial cells and mice exposed to house dust mite prior to poly (I: C) treatment exhibited a reduced anti-viral interferon response, including reduced IFN-β, IFN-λ, TLR3, RIG-1, MDA5, and IRF-3 levels. Furthermore, heat inactivation of house dust mites partially restored the TLR3-induced interferon response (60). Proteolytic allergens such as house dust mite trigger ROS production, consequently, oxidative stress and enhance the weakening of antioxidant responses by DNA damage (61, 62). Taken together, these data indicate that the anti-viral interferon production enhanced by PRRs stimulation could be attenuated by oxidative stress or by altering the antioxidant response. In contrast, other studies have shown that pretreatment with H_2_O_2_ enhanced the upregulated expression of TLR3 and downregulated IRF3 (50). In primary human airway epithelial cells treated with poly (I: C), the up-regulation of TLR3 levels was not inhibited by pretreatment with H_2_O_2_ (63). Furthermore, comparable studies are required to clarify these controversial issues.

Initially, Nrf2 was mainly considered a regulator of redox homeostasis (64). However, recent research has implicated Nrf2 as an important regulator of innate immunity (65). The current results indicated that the secretion of anti-viral interferon was markedly potentiated in cells pretreated with sulforaphane, an activator of Nrf2. However, Nrf2-knockdown cells showed attenuation of anti-viral interferon secretion even after RV-16 inoculation or poly (I: C) stimulation. Based on our data, Nrf2 may act as a modulator of innate immunity in sinonasal mucosa.

In conclusion, this study provides evidence that the levels of H_2_O_2_ increase in the nasal secretions of patients with CRS. Therefore, in the present study, we used H_2_O_2_ as an oxidative stressor to elucidate the role of oxidative stress in the regulation of anti-viral interferon production in the sinonasal mucosa. Our data indicated that the pretreatment with H_2_O_2_ attenuates RV 16- and poly (I: C)-induced secretion of anti-viral interferons. The expression levels of PRRs and IRF3 were also down-regulated in cells pretreated with H_2_O_2._ In addition, the secretion of anti-viral interferons was reduced by deletion of the Nrf2, but potentiated by the Nrf2 activator. Therefore, the present study suggests that oxidative stress may inhibit the production of RV-induced anti-viral interferons in the sinonasal mucosa.

## Data availability statement

The datasets presented in this study can be found in online repositories. The names of the repository/repositories and accession number(s) can be found in the article/**Supplementary Material**.

## Ethics statement

The studies involving human participants were reviewed and approved by Korea University Hospital Ethics Committee. The patients/participants provided their written informed consent to participate in this study.

## Author contributions

SaHL onceived and designed the study and performed experiments. MH and TK performed the collection of samples and experiments and analyzed data. TL, DL, JP, SeHL, TK erformed the collection of samples, the performance of experiments, analyzed data and contributed to data visualization. All authors wrote the manuscript and approved the final manuscript.

## Supplementary material

Supplementary Figure 1 The graphs **(A, B)** indicate the RV 16 replication rates in each groups of cultured cells which were represented in **Figures 1A**, **2B**.

Supplementary Figure 2 The graphs **(A–C)** indicate the RV 16 replication rates in each groups of cultured cells which were represented in **Figures 3C**.

Supplementary Figure 3 The graphs **(A–C)** indicate the RV 16 replication rates in each groups of cultured cells which were represented in **Figures 4A, C, E**.

Supplementary Figure 4 The graphs **(A–D)** indicate the RV 16 replication rates in each groups of cultured cells which were represented in **Figures 5A, C, E**, **6A**.

Supplementary Figure 5 The graphs **(A–F)** indicate the RV 16 replication rates in each groups of cultured cells which were represented in **Figures 7A–F**.

Supplementary Figure 6 The expression levels of SOD1 **(A, C)** and SOD2 **(B, D)** in cultured sinonasal epithelial cells pretreated with H_2_O_2_ at 50, 100, and 150 uM and then, followed by RV 16 infection **(A, B)** and poly (I: C) treatment **(C, D)**, which was evaluated by RT– qPCR. Data are mean ± SEM from 7 different epithelial donors. Control indicates non-treated normal epithelial cells. Rhino indicates epithelial cells infected with RV 16. H_2_O_2_ +rhino indicates the epithelial cells pretreated with H_2_O_2_ at 50, 100, and 150 uM followed by RV 16 infection. NAC+ H_2_O_2_ +rhino indicates the cells which were pretreated with NAC at 5 mM for 1 h and then followed by H_2_O_2_ treatment at 100 uM and subsequently infected with RV 16. Poly (I: C) indicates epithelial cells treated with poly (I: C). H_2_O_2_ + poly (I: C) indicates the epithelial cells pretreated with H_2_O_2_ at 50, 100, and 150 uM followed by poly (I: C) treatment. NAC+ H_2_O_2_ + poly (I: C) indicates the cells which was pretreated with NAC at 5 mM for 1 h and then followed by H_2_O_2_ treatment at 100 uM and subsequently treated with poly (I: C). Rhino indicates RV 16. NAC indicates N-acetyl-L-cysteine.

Supplementary Figure 7 The expression level of IFN-β **(A, B)**, IFN-λ1 **(C, D)**, and IFN-λ2 (E, F) mRNA in sinonasal epithelial cells pretreated with H_2_O_2_ at 50, 100, and 150 uM and then, followed by RV 16 infection **(A, C, E)** or poly (I: C) treatment **(B, D, F)** which were evaluated by RT– qPCR. Data are mean ± SEM from 7 different epithelial donors. Control indicates non-treated normal epithelial cells. Rhino indicates epithelial cells infected with RV 16. H_2_O_2_ + rhino indicates the epithelial cells pretreated with H_2_O_2_ at 50, 100, and 150 uM followed by RV 16 infection. NAC+ H_2_O_2_ +rhino indicates the cells which were pretreated with NAC at 5 mM for 1 h and then followed by H_2_O_2_ treatment at 100 uM and subsequently infected with RV 16. Poly (I: C) indicates epithelial cells treated with poly (I: C). H_2_O_2_ + poly (I: C) indicates the epithelial cells pretreated with H_2_O_2_ at 50, 100, and 150 uM followed by poly (I: C) treatment. NAC+ H_2_O_2_ + poly (I: C) indicates the cells which was pretreated with NAC at 5 mM for 1 h and then followed by H_2_O_2_ treatment at 100 uM and subsequently treated with poly (I: C). Rhino indicates RV 16. NAC indicates N-acetyl-L-cysteine.

Supplementary Figure 8 The expression level of viperin **(A, B)**, Mx **(C, D)**, and OAS **(E, F)** mRNA in sinonasal epithelial cells pretreated with H_2_O_2_ at 50, 100, and 150 uM and then, followed by RV 16 infection **(A, C, E)** or poly (I: C) treatment **(B, D, F)** which were evaluated with RT-qPCR. Data are mean ± SEM from 7 different epithelial donors. Control indicates non-treated normal epithelial cells. Rhino indicates epithelial cells infected with RV 16. H_2_O_2_ + rhino indicates the epithelial cells pretreated with H_2_O_2_ at 50, 100, and 150 uM followed by RV 16 infection. NAC+ H_2_O_2_ +rhino indicates the cells which were pretreated with NAC at 5 mM for 1 h and then followed by H_2_O_2_ treatment at 100 uM and subsequently infected with RV 16. Poly (I: C) indicates epithelial cells treated with poly (I: C). H_2_O_2_ + poly (I: C) indicates the epithelial cells pretreated with H_2_O_2_ at 50, 100, and 150 uM followed by poly (I: C) treatment. NAC+ H_2_O_2_ + poly (I: C) indicates the cells which was pretreated with NAC at 5 mM for 1 h and then followed by H_2_O_2_ treatment at 100 uM and subsequently treated with poly (I: C).

Supplementary Figure 9 The expression level of TLR3 (A, B), RIG-I **(C, D)**, and MDA5 mRNA **(E, F)** in sinonasal epithelial cells pretreated with H_2_O_2_ at 50, 100, and 150 uM and then, followed by RV 16 infection **(A, C, E)** or poly (I: C) treatment **(B, D, F)** which were evaluated with RT-qPCR. Data are mean ± SEM from 7 different epithelial donors. Control indicates non-treated normal epithelial cells. Rhino indicates epithelial cells infected with RV 16. H_2_O_2_ + rhino indicates the epithelial cells pretreated with H_2_O_2_ at 50, 100, and 150 uM followed by RV 16 infection. NAC+ H_2_O_2_ +rhino indicates the cells which were pretreated with NAC at 5 mM for 1 h and then followed by H_2_O_2_ treatment at 100 uM and subsequently infected with RV 16. Poly (I: C) indicates epithelial cells treated with poly (I: C). H_2_O_2_ + poly (I: C) indicates the epithelial cells pretreated with H_2_O_2_ at 50, 100, and 150 uM followed by poly (I: C) treatment. NAC+ H_2_O_2_ + poly (I: C) indicates the cells which was pretreated with NAC at 5 mM for 1 h and then followed by H_2_O_2_ treatment at 100 uM and subsequently treated with poly (I: C).

Supplementary Figure 10 The expression of IFN-β (A), IFN-λ1 **(C)**, IFN-λ2 **(E)** viperin **(B)**, Mx **(D)**, and OAS **(F)** mRNA in sinonasal epithelial cells transfected with Nrf2 siRNA and pretreated with sulforaphane and then, followed by RV 16 infection which were evaluated with RE-qPCR. Data are mean ± SEM from 7 different epithelial donors. Control indicates non-treated normal epithelial cells. Rhino indicates epithelial cells infected with RV 16. Sulforaphane + rhino indicates the epithelial cells pretreated with sulforaphane at 5 uM followed by RV 16 infection. Nrf2siRNA + rhino indicates the cells transfected with Nrf2 siRNA followed by infection with RV 16.

Supplementary Figure 11 The expression of IFN-β **(A)**, IFN-λ1 **(C)**, IFN-λ2 **(E)**, viperin **(B)**, Mx **(D)**, and OAS **(F)** mRNA in sinonasal epithelial cells transfected with Nrf2 siRNA and pretreated with sulforaphane and then, followed by poly (I: C) treatment which was evaluated with RE-qPCR. Data are mean ± SEM from 7 different epithelial donors. Control indicates non-treated normal epithelial cells. Poly (I: C) indicates epithelial cells treated with Poly (I: C). Sulforaphane + Poly (I: C) indicates the epithelial cells pretreated with sulforaphane at 5 uM followed by Poly (I: C) treatment. Nrf2siRNA + Poly (I:C) indicates the cells transfected with Nrf2 siRNA followed by treatment with Poly (I: C).
