# Supplementary figures and images for: Hydrogen peroxide attenuates rhinovirus-induced anti-viral interferon secretion in sinonasal epithelial cells
Source: Front Immunol. 2023 Feb 13;14:1086381. doi: 10.3389/fimmu.2023.1086381 (PMC9968966; doi:10.3389/fimmu.2023.1086381)

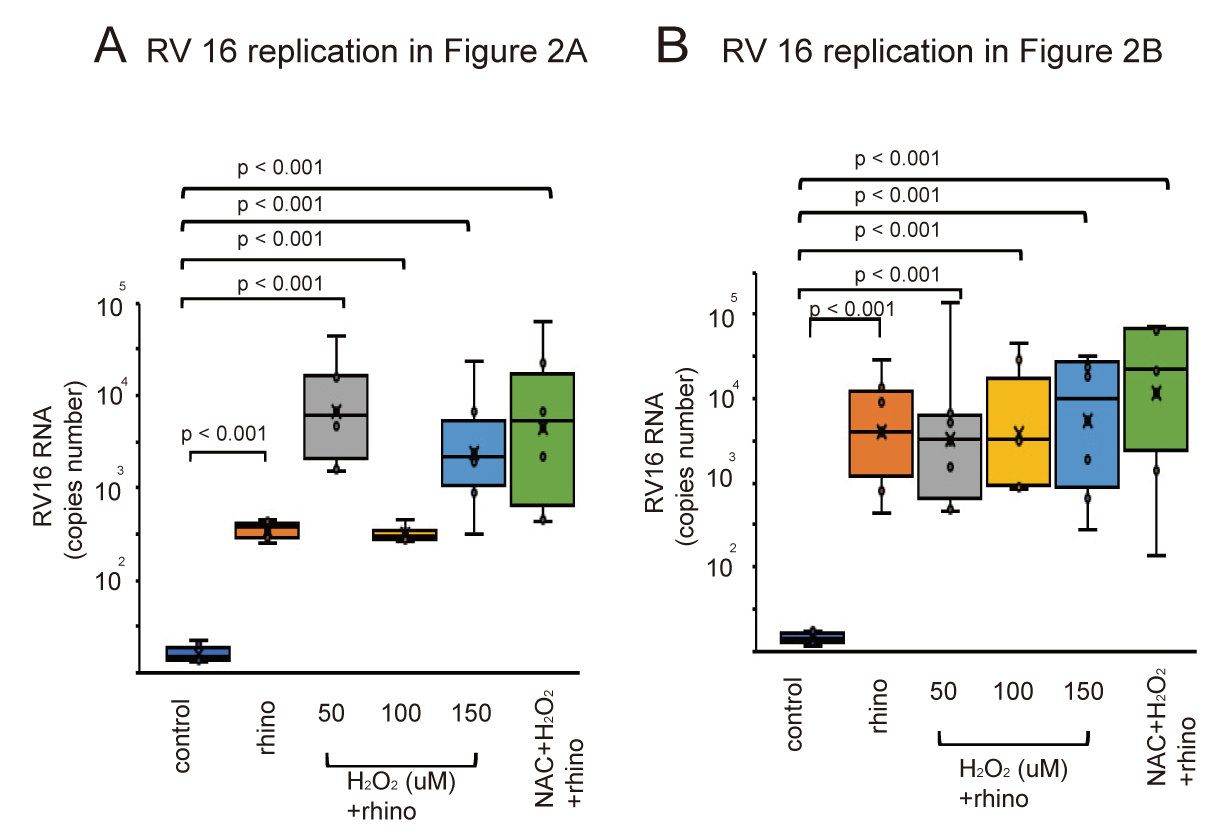

Supplement: Supplementary Figure 1 — The graphs (A, B) indicate the RV 16 replication rates in each groups of cultured cells which were represented in Figures 1A , 2B . [file Image_1.tif]

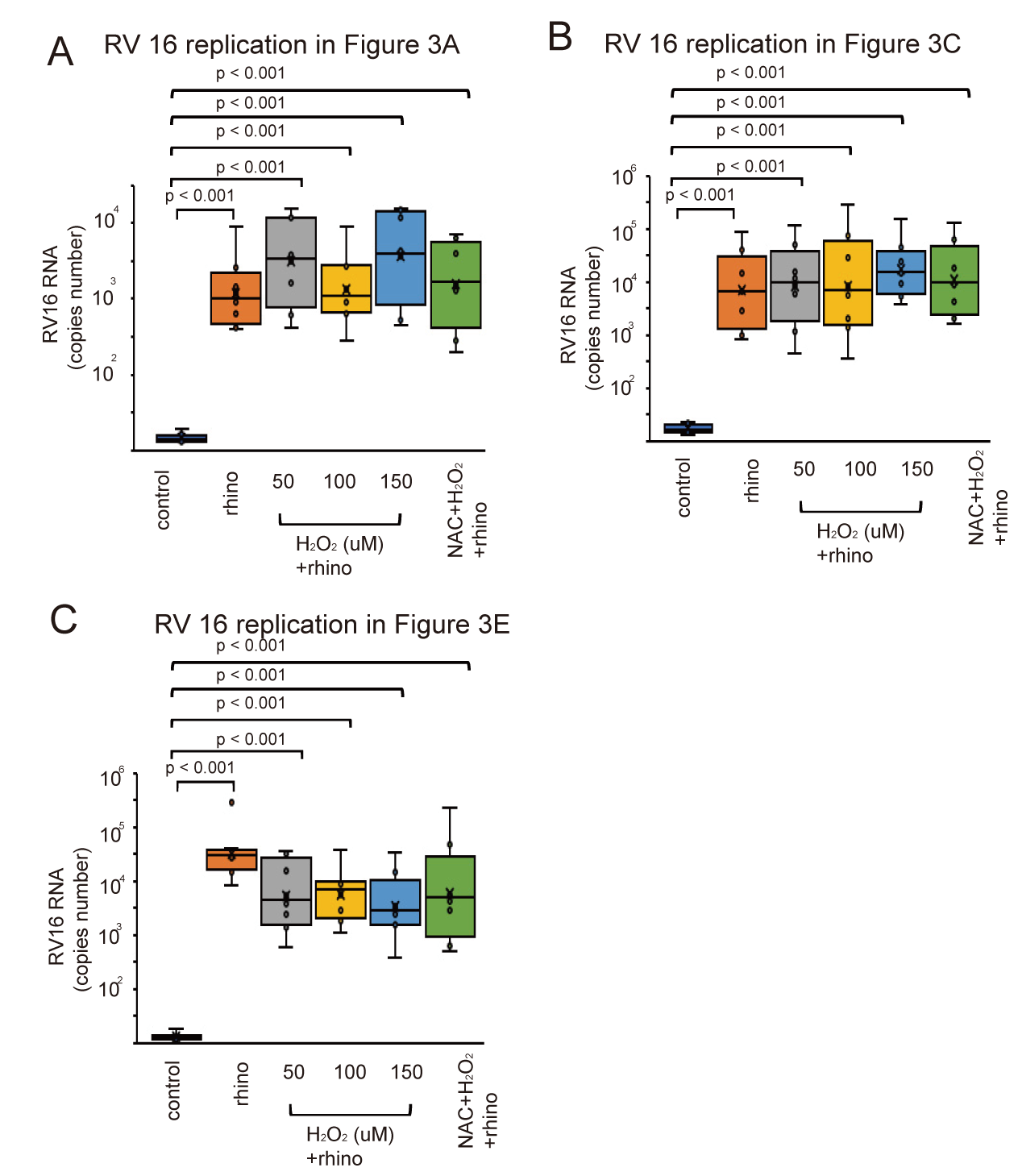

Supplement: Supplementary Figure 2 — The graphs (A–C) indicate the RV 16 replication rates in each groups of cultured cells which were represented in Figures 3C . [file Image_2.tif]

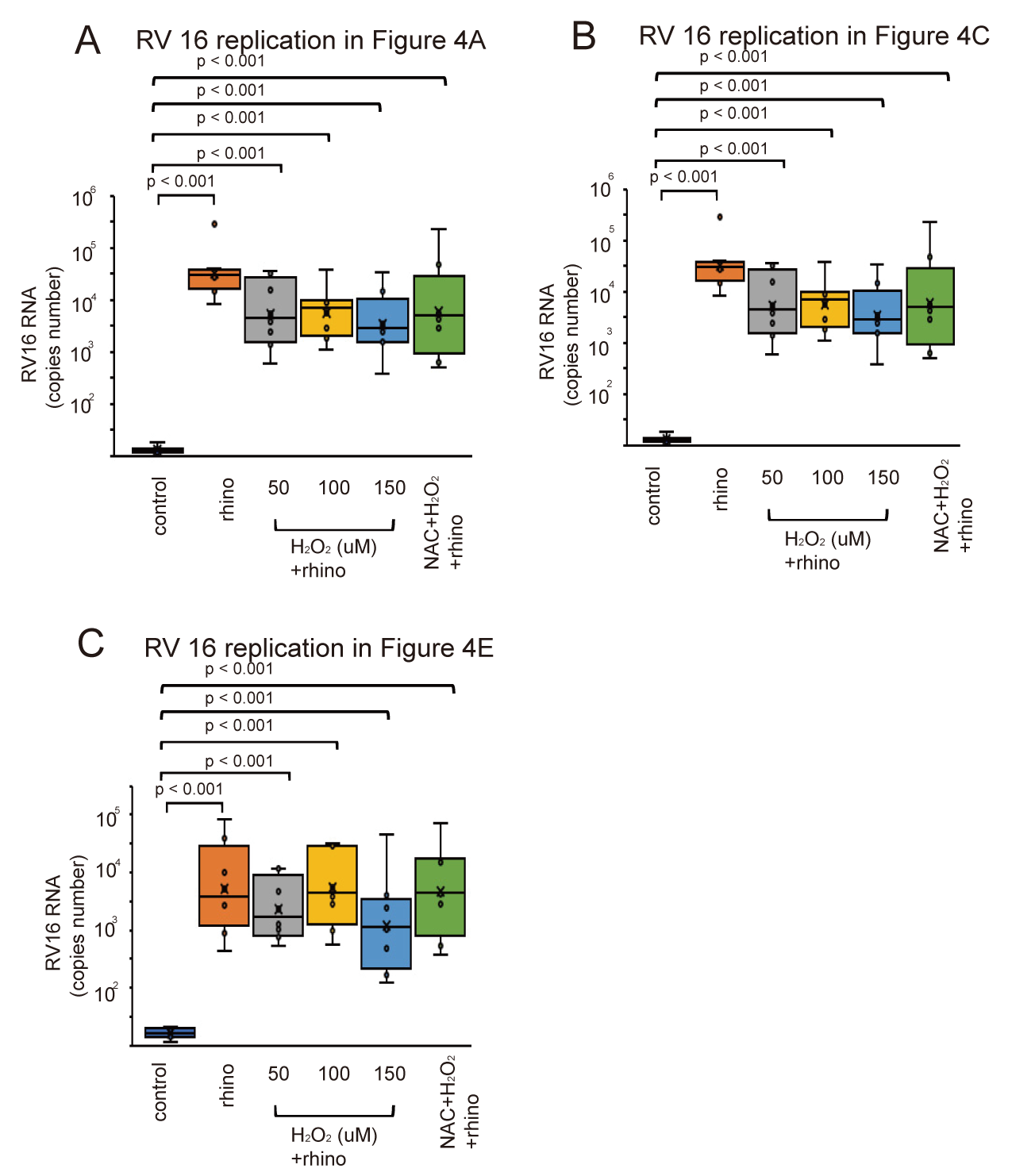

Supplement: Supplementary Figure 3 — The graphs (A–C) indicate the RV 16 replication rates in each groups of cultured cells which were represented in Figures 4A, C, E . [file Image_3.tif]

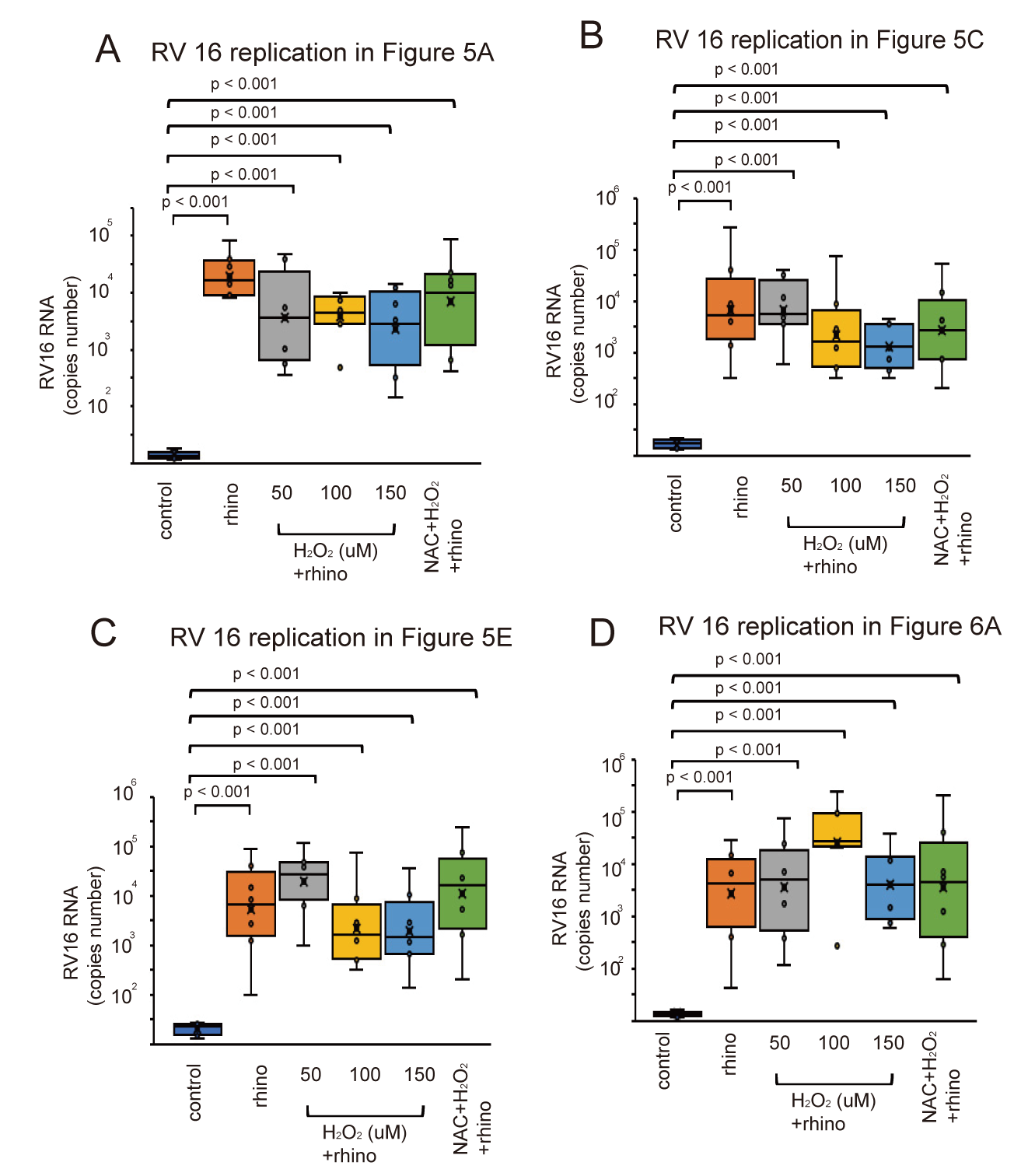

Supplement: Supplementary Figure 4 — The graphs (A–D) indicate the RV 16 replication rates in each groups of cultured cells which were represented in Figures 5A, C, E , 6A . [file Image_4.tif]

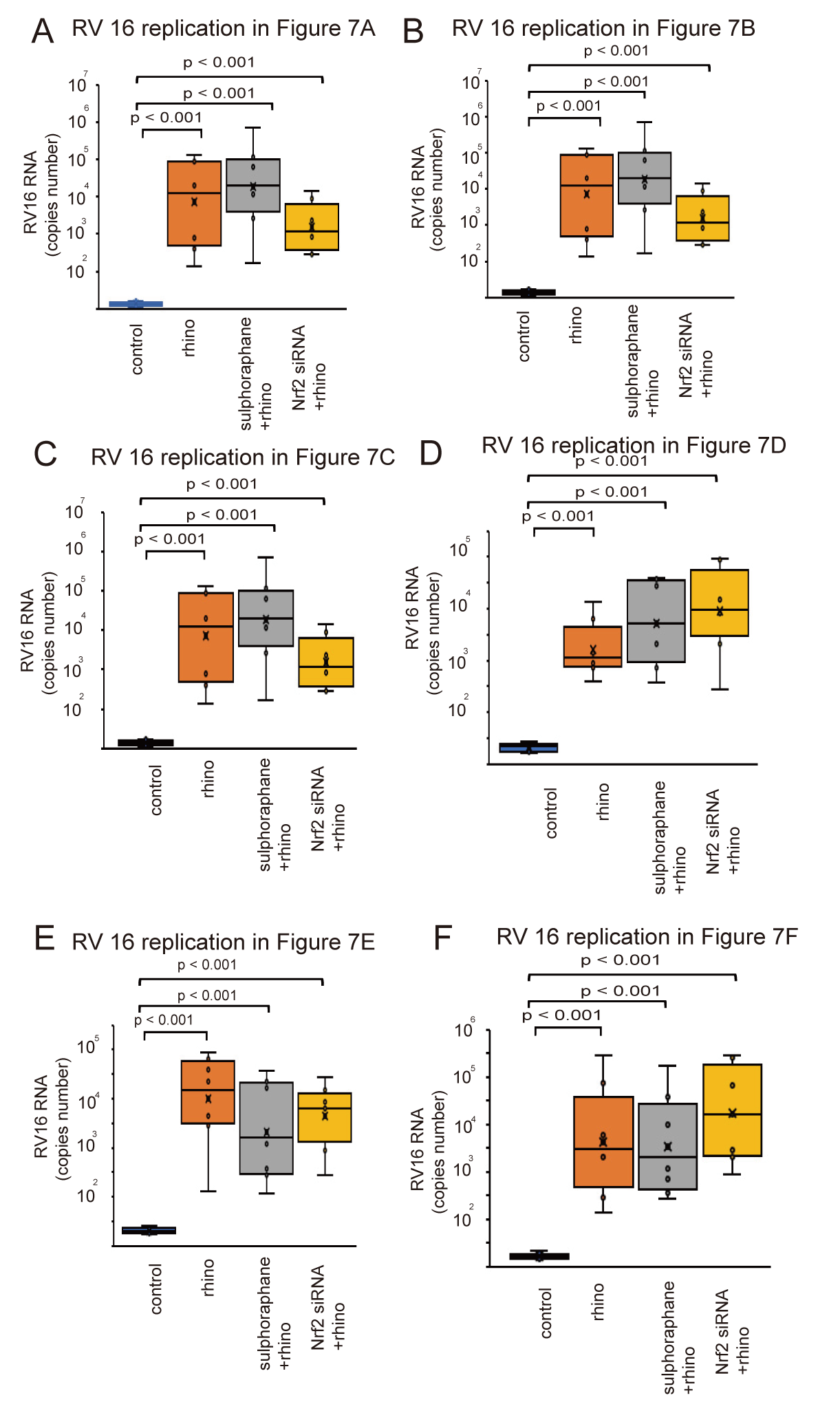

Supplement: Supplementary Figure 5 — The graphs (A–F) indicate the RV 16 replication rates in each groups of cultured cells which were represented in Figures 7A–F . [file Image_5.tif]

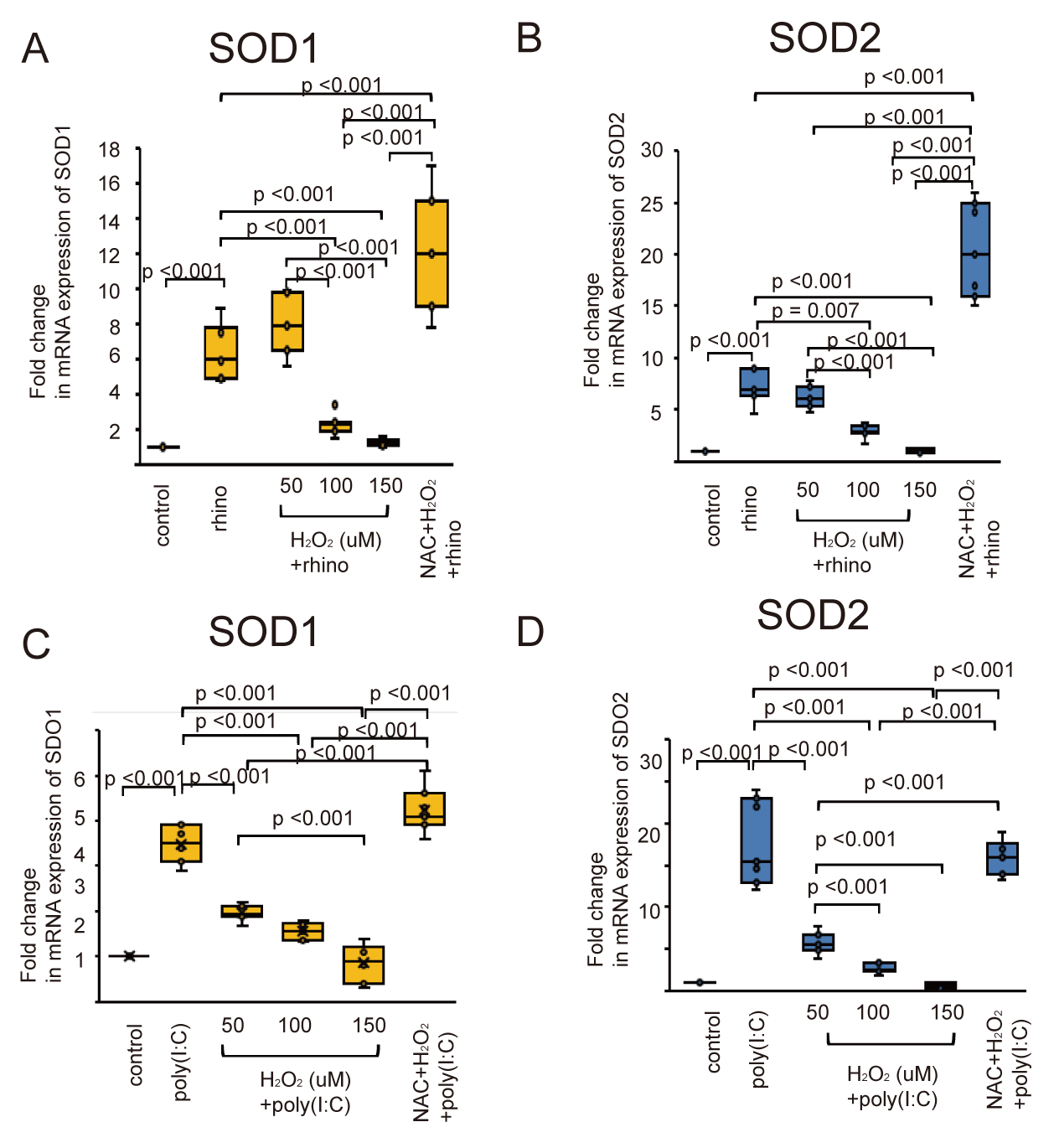

Supplement: Supplementary Figure 6 — The expression levels of SOD1 (A, C) and SOD2 (B, D) in cultured sinonasal epithelial cells pretreated with H2O2 at 50, 100, and 150 uM and then, followed by RV 16 infection (A, B) and poly (I: C) treatment (C, D), which was evaluated by RT– qPCR. Data are mean ± SEM from 7 different epithelial donors. Control indicates non-treated normal epithelial cells. Rhino indicates epithelial cells infected with RV 16. H2O2 +rhino indicates the epithelial cells pretreated with H2O2 at 50, 100, and 150 uM followed by RV 16 infection. NAC+ H2O2 +rhino indicates the cells which were pretreated with NAC at 5 mM for 1 h and then followed by H2O2 treatment at 100 uM and subsequently infected with RV 16. Poly (I: C) indicates epithelial cells treated with poly (I: C). H2O2 + poly (I: C) indicates the epithelial cells pretreated with H2O2 at 50, 100, and 150 uM followed by poly (I: C) treatment. NAC+ H2O2 + poly (I: C) indicates the cells which was pretreated with NAC at 5 mM for 1 h and then followed by H2O2 treatment at 100 uM and subsequently treated with poly (I: C). Rhino indicates RV 16. NAC indicates N-acetyl-L-cysteine. [file Image_6.tif]

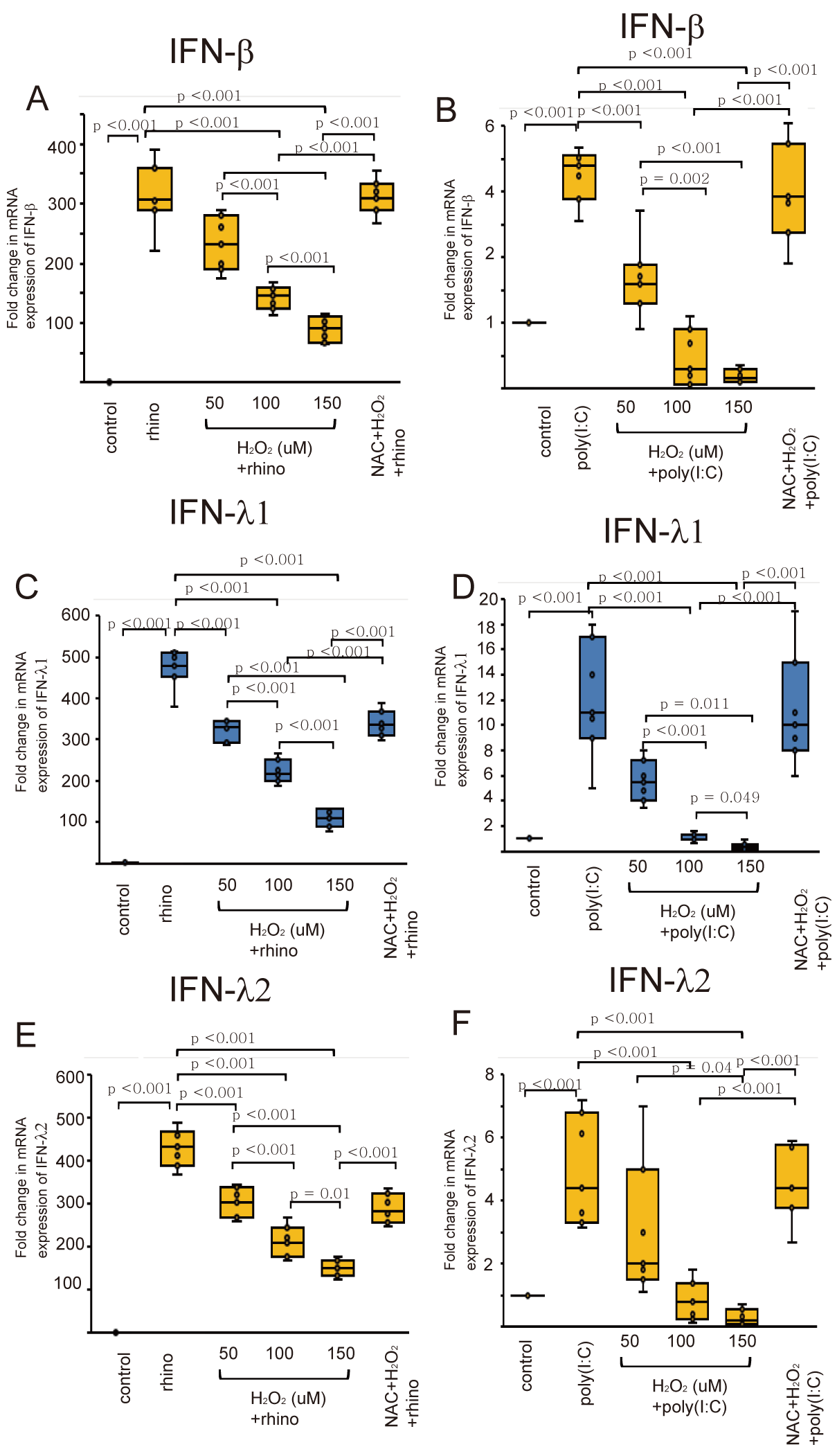

Supplement: Supplementary Figure 7 — The expression level of IFN-β (A, B), IFN-λ1 (C, D), and IFN-λ2 (E, F) mRNA in sinonasal epithelial cells pretreated with H2O2 at 50, 100, and 150 uM and then, followed by RV 16 infection (A, C, E) or poly (I: C) treatment (B, D, F) which were evaluated by RT– qPCR. Data are mean ± SEM from 7 different epithelial donors. Control indicates non-treated normal epithelial cells. Rhino indicates epithelial cells infected with RV 16. H2O2 + rhino indicates the epithelial cells pretreated with H2O2 at 50, 100, and 150 uM followed by RV 16 infection. NAC+ H2O2 +rhino indicates the cells which were pretreated with NAC at 5 mM for 1 h and then followed by H2O2 treatment at 100 uM and subsequently infected with RV 16. Poly (I: C) indicates epithelial cells treated with poly (I: C). H2O2 + poly (I: C) indicates the epithelial cells pretreated with H2O2 at 50, 100, and 150 uM followed by poly (I: C) treatment. NAC+ H2O2 + poly (I: C) indicates the cells which was pretreated with NAC at 5 mM for 1 h and then followed by H2O2 treatment at 100 uM and subsequently treated with poly (I: C). Rhino indicates RV 16. NAC indicates N-acetyl-L-cysteine. [file Image_7.tif]

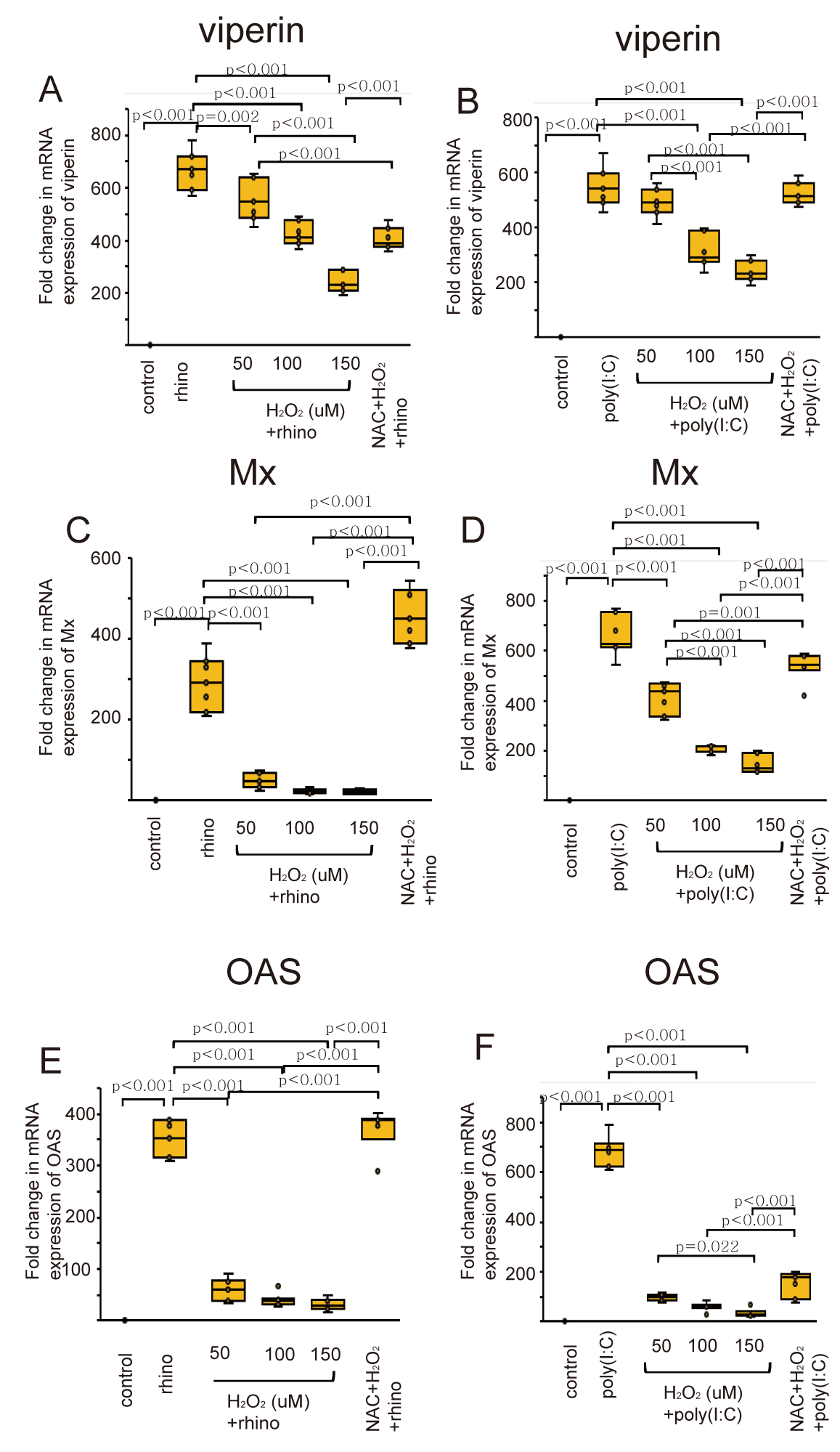

Supplement: Supplementary Figure 8 — The expression level of viperin (A, B), Mx (C, D), and OAS (E, F) mRNA in sinonasal epithelial cells pretreated with H2O2 at 50, 100, and 150 uM and then, followed by RV 16 infection (A, C, E) or poly (I: C) treatment (B, D, F) which were evaluated with RT-qPCR. Data are mean ± SEM from 7 different epithelial donors. Control indicates non-treated normal epithelial cells. Rhino indicates epithelial cells infected with RV 16. H2O2 + rhino indicates the epithelial cells pretreated with H2O2 at 50, 100, and 150 uM followed by RV 16 infection. NAC+ H2O2 +rhino indicates the cells which were pretreated with NAC at 5 mM for 1 h and then followed by H2O2 treatment at 100 uM and subsequently infected with RV 16. Poly (I: C) indicates epithelial cells treated with poly (I: C). H2O2 + poly (I: C) indicates the epithelial cells pretreated with H2O2 at 50, 100, and 150 uM followed by poly (I: C) treatment. NAC+ H2O2 + poly (I: C) indicates the cells which was pretreated with NAC at 5 mM for 1 h and then followed by H2O2 treatment at 100 uM and subsequently treated with poly (I: C). [file Image_8.tif]

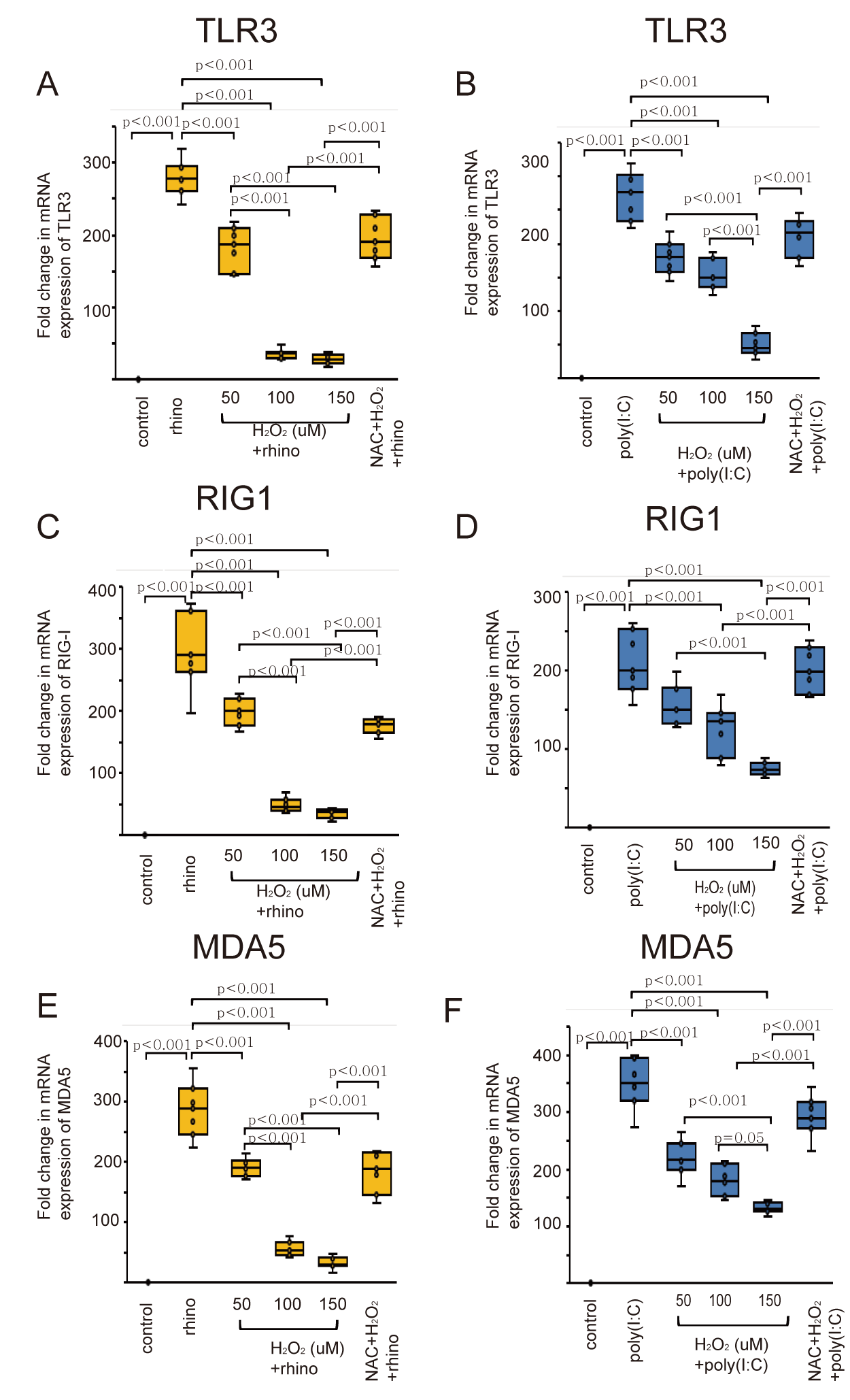

Supplement: Supplementary Figure 9 — The expression level of TLR3 (A, B), RIG-I (C, D), and MDA5 mRNA (E, F) in sinonasal epithelial cells pretreated with H2O2 at 50, 100, and 150 uM and then, followed by RV 16 infection (A, C, E) or poly (I: C) treatment (B, D, F) which were evaluated with RT-qPCR. Data are mean ± SEM from 7 different epithelial donors. Control indicates non-treated normal epithelial cells. Rhino indicates epithelial cells infected with RV 16. H2O2 + rhino indicates the epithelial cells pretreated with H2O2 at 50, 100, and 150 uM followed by RV 16 infection. NAC+ H2O2 +rhino indicates the cells which were pretreated with NAC at 5 mM for 1 h and then followed by H2O2 treatment at 100 uM and subsequently infected with RV 16. Poly (I: C) indicates epithelial cells treated with poly (I: C). H2O2 + poly (I: C) indicates the epithelial cells pretreated with H2O2 at 50, 100, and 150 uM followed by poly (I: C) treatment. NAC+ H2O2 + poly (I: C) indicates the cells which was pretreated with NAC at 5 mM for 1 h and then followed by H2O2 treatment at 100 uM and subsequently treated with poly (I: C). [file Image_9.tif]

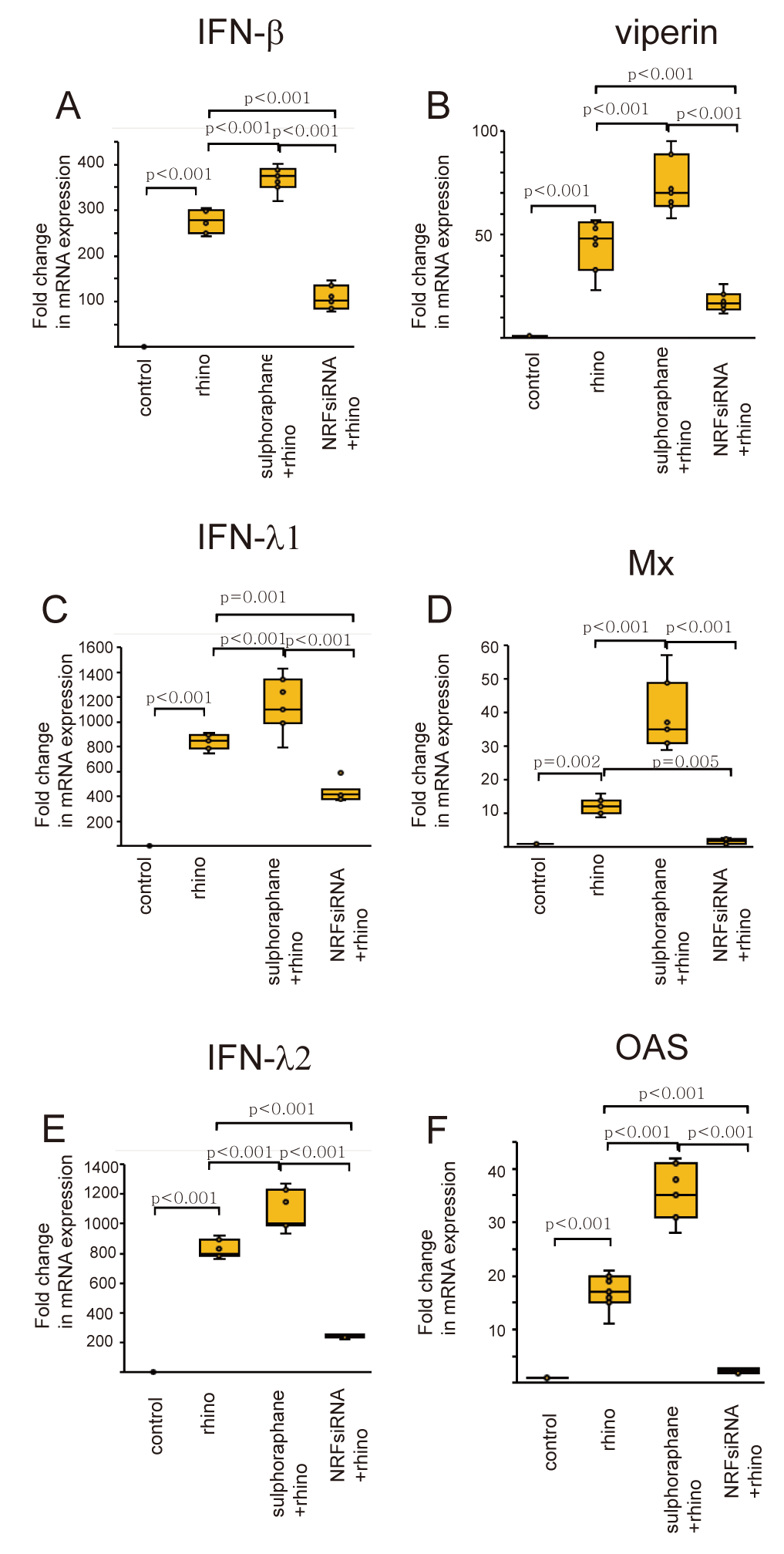

Supplement: Supplementary Figure 10 — The expression of IFN-β (A), IFN-λ1 (C), IFN-λ2 (E) viperin (B), Mx (D), and OAS (F) mRNA in sinonasal epithelial cells transfected with Nrf2 siRNA and pretreated with sulforaphane and then, followed by RV 16 infection which were evaluated with RE-qPCR. Data are mean ± SEM from 7 different epithelial donors. Control indicates non-treated normal epithelial cells. Rhino indicates epithelial cells infected with RV 16. Sulforaphane + rhino indicates the epithelial cells pretreated with sulforaphane at 5 uM followed by RV 16 infection. Nrf2siRNA + rhino indicates the cells transfected with Nrf2 siRNA followed by infection with RV 16. [file Image_10.tif]

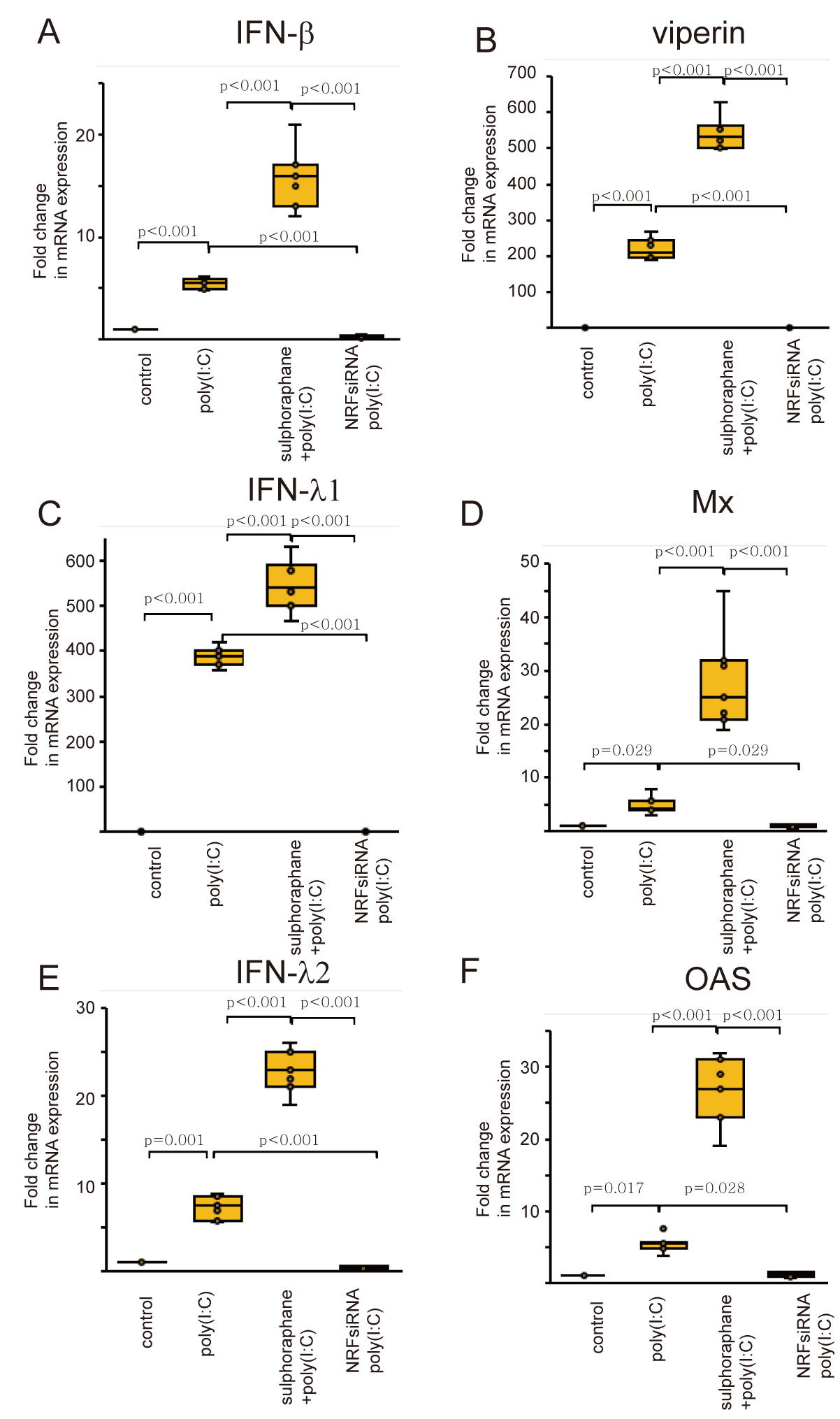

Supplement: Supplementary Figure 11 — The expression of IFN-β (A), IFN-λ1 (C), IFN-λ2 (E), viperin (B), Mx (D), and OAS (F) mRNA in sinonasal epithelial cells transfected with Nrf2 siRNA and pretreated with sulforaphane and then, followed by poly (I: C) treatment which was evaluated with RE-qPCR. Data are mean ± SEM from 7 different epithelial donors. Control indicates non-treated normal epithelial cells. Poly (I: C) indicates epithelial cells treated with Poly (I: C). Sulforaphane + Poly (I: C) indicates the epithelial cells pretreated with sulforaphane at 5 uM followed by Poly (I: C) treatment. Nrf2siRNA + Poly (I:C) indicates the cells transfected with Nrf2 siRNA followed by treatment with Poly (I: C). [file Image_11.tif]
