# Supplementary material for: Hydrogen peroxide attenuates rhinovirus-induced anti-viral interferon secretion in sinonasal epithelial cells
Source: Front Immunol. 2023 Feb 13;14:1086381. doi: 10.3389/fimmu.2023.1086381 (PMC9968966; doi:10.3389/fimmu.2023.1086381)
Supplement: Supplementary file 12 [file Table_1.docx]

Table 1. Characteristics of the study group

Healthy control subjects CRSsNP CRSwNP

Septal deviation blowout fracture

(n = 50) (n=15) (n = 23) (n = 26)

Sex

Male 35 13 13 16

Female 15 2 10 10

Mean age (Y) 31.13±10.02 35.45±14.67 39.12±17.76 43.76 ± 14.64

SNOT-20

Score* 8.14 ± 2.58 7.12±1.45 34.23 ± 5.54 36.63± 7.8

Computed

tomographic

grade* 1.45 ± 0.38 1.23± 0.46 6.34 ± 1.57 15.5 ± 2.12

Endoscopy

Score* 0 0 4.65±1.65 8.43±2.14

*SNOT-20 score, computed tomographic grades, and endoscopy score in patients with CRSsNP and patients with CRSwNP are higher than healthy control subjects (p<.05). However, there are no significant differences in these scores between patients with CRSsNP and those with CRSwNP. SNOT-20 score; the average 20-item Sino-Nasal Outcome Test score.
