# Supplementary material for: Hydrogen peroxide attenuates rhinovirus-induced anti-viral interferon secretion in sinonasal epithelial cells
Source: Front Immunol. 2023 Feb 13;14:1086381. doi: 10.3389/fimmu.2023.1086381 (PMC9968966; doi:10.3389/fimmu.2023.1086381)
Supplement: Supplementary file 13 [file Table_2.docx]

Table 2. Primers used for real-time qPCR analysis

Primer Sequence

IFN-β S:5′- GCACAACAGGTAGTAGGCGA-3′ AS: 5′- TGGAAAGAGCTGTCGTGGAG -3′

IFN-λ1 S:5′- GGTGACTTTGGTGCTAGGCT-3′ AS: 5′- GGCCTTCTTGAAGCTCGCTA -3′

IFN-λ2 S:5′- GTGACAGCCTCAGAGTGTTTCT-3′ AS: 5′- AACTGCTCCAGTCACGGTCA -3′

Viperin S:5′- GGCAGTGCTTGCATTGCTTT-3′ AS: 5′- CTTATGCCAACCCAGTGTAACG -3′

Mx S:5′- CAGCTCAGGGGCTTTGGAAT-3′ AS: 5′- CCTTGGAATGGTGGCTGGAT -3′

OAS S:5′- GCTGAGGCCTGGCTGAATTA -3′ AS: 5′- CAGTCCTCTTCTGCCTGTGG -3′

TLR 3 S:5′- AGTGCCGTCTATTTGCCACA -3′ AS: 5′- GCATCCCAAAGGGCAAAAGG -3′

RIG-1 S:5′- AGAGCACTTGTGGACGCTTT -3′ AS: 5′- TGTTTTGCCACGTCCAGTCA -3′

MDA5 S: 5′- TTGGACTCGGGAATTCGTGG -3′ AS: 5′- AGCTCAGGGTTCATGTAGCG -3′

Nrf2 S: 5′- AGCGACGGAAAGAGTATGAGC -3′ AS: 5′- TGGGCAACCTGTCTCTTCAT -3′

GAPDH S:5′- CCACATCGCTCAGACACCAT -3′ AS: 5′- AGTTAAGAACAGCCCTGGTGA -3′
